# Plant miRNAs influence soil bacterial growth and amino acid uptake, restructuring community composition

**DOI:** 10.1093/ismeco/ycaf206

**Published:** 2025-11-08

**Authors:** Jessica A Dozois, Marc-Antoine Duchesne, Katel Hallaf, Julien Tremblay, Étienne Yergeau

**Affiliations:** Institut National de la Recherche Scientifique, Centre Armand-Frappier Santé Biotechnologie, Laval, QC, H7V 1B7, Canada; Institut National de la Recherche Scientifique, Centre Armand-Frappier Santé Biotechnologie, Laval, QC, H7V 1B7, Canada; Institut National de la Recherche Scientifique, Centre Armand-Frappier Santé Biotechnologie, Laval, QC, H7V 1B7, Canada; Institut National de la Recherche Scientifique, Centre Armand-Frappier Santé Biotechnologie, Laval, QC, H7V 1B7, Canada; Institut National de la Recherche Scientifique, Centre Armand-Frappier Santé Biotechnologie, Laval, QC, H7V 1B7, Canada

**Keywords:** plant miRNAs, amino acids, nitrogen, soil bacterial communities, plant-microbe interactions

## Abstract

Plants and microbes use many strategies to acquire soil amino acids. Recent findings suggest that genes related to amino acid metabolism and transport are influenced by plant miRNAs. Here, we first show that *Arabidopsis* modifies its root miRNA content when fertilized with a mixture of 17 amino acids. The miRNAs that responded to amino acid fertilization and other rhizosphere-abundant miRNAs were applied to a simplified soil community, grown with diverse amino acid sources, to test if they interfered with microbial community growth, community composition, and amino acid consumption. Plant miRNAs affected the community’s growth in over 70% of the amino acid sources. The impact of plant miRNAs also depended on the N source supplied to the microbial community, with the strongest effect observed with L-lysine. Specifically, ath-miR159a reduced the microbial consumption of L-lysine, further supporting that plant miRNAs can influence microbial amino acid uptake. Plant miRNAs also strongly affected the relative abundance of specific bacterial taxa, which we subsequently isolated. These community shifts were explained by the subtle but robust impact of plant miRNAs on isolates' growth and, for two out of three isolates, on amino acid consumption. Surprisingly, while plant miRNAs inhibited amino acid consumption at both the community and isolate levels, the effects of plant miRNAs were mostly positive. Our results suggest that rhizospheric plant miRNAs might have a role in modulating the amino acid consumption of soil bacteria which reshapes the community, but not necessarily in a competitive framework.

## Graphical Abstract

**Graphical Abstract:**
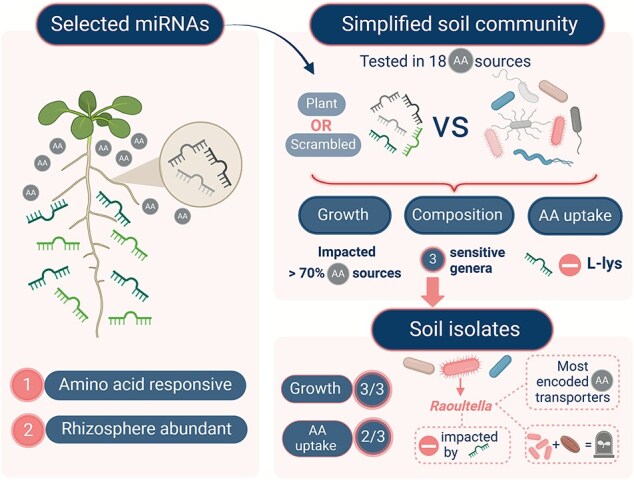

## Introduction

Nitrogen (N) is one of the most limiting nutrients in terrestrial ecosystems—hence the subject of a fierce competition between plants and microorganisms [1]. Recent findings from our team revealed that plant miRNAs impact the expression of many bacterial genes related to N, including genes related to amino acid uptake and metabolism [2]. Although the concentration of amino acids in the soil is rather low (~20 μM) [3], the pool is rapidly renewed within minutes to hours [4–6]. The uptake of amino acids by microbes exceeds, by a factor >8, the rate at which they convert organic N to ammonium and nitrate [7]. When amino acid concentrations are high, plants uptake less nitrate [8–10]. Amino acids are thus sought after by microbes and plants. To impair plant amino acid uptake and increase the efflux of amino acids from the roots, microbes such as *Fusarium* can produce zearalenone while *Pseudomonas*, *Chromobacterium* can produce 2,4-diacetylphloroglucinol and isolates belonging to the phyla *Actinomycetota* and *Pseudomonadota* can produce phenazine [11–13]. To better colonize the root-soil interface, cytokinin-producing bacteria, like *Bacillus subtilis*, can also increase amino acid rhizodeposition [14]. Although microbes uptake soil amino acids more rapidly, plants have high affinity transporters for amino acids [15] and are competitive in environmental conditions such as soil acidification [16]. Bacterial sRNAs have been shown to modify amino acid metabolism in ways that strongly optimize root colonization by a rhizobial symbiont [17]. Plants also stimulate microbes to depolymerize soil organic nitrogen and, in time, uptake N released from the microbial necromass [18]. Some plants directly influence soil N-cycling by producing compounds that inhibit microbes from performing nitrification and denitrification [19–25]. Some plant secondary metabolites may interfere with bacterial amino acid uptake [26–28]. However, if confirmed, plant miRNAs would be the first RNA molecule that plants use to interfere with microbial amino acid uptake. Within the plant, specific miRNAs regulate N metabolism and respond to different exogenous N treatments [29–35], but their effect on the microbial community is not known.

The effectiveness of RNA interference (RNAi) across kingdoms has been shown for two miRNAs in cotton plants (*Gossypium*) that inhibited the virulence of a fungal pathogen (*Verticillium dahliae*) [36]. Plant miRNAs and small RNAs are also exchanged from plants to animals [37–43] where they have been found to affect host cell activity and regulate the gut microbiota. This RNA cross-talk has also been shown from plants to plants [44, 45], from plants to phytopathogens [36, 46], from microbial pathogens to plants [47, 48] and from plant symbionts to plants [49, 50]. The ability of miRNAs to interfere with mRNA translation relies on miRNA-mRNA base pairing. In animals, the seed region of the miRNA (2–7 nucleotides at the 5′ end) is important for target recognition which often occurs in the 3′ untranslated region (3’ UTR) [51, 52]. In plants, the miRNA-mRNA pairing is fully or nearly complementary and the binding sites are usually found in the coding sequence (CDS) [52]. In bacteria, the length of sRNAs range from 40–500 nt and complementarity is variable from perfect matches (cis-acting sRNAs) to imperfect matches (trans-acting sRNAs) to sRNAs that even bind to proteins [53]. The structure of these lengthier sRNAs also plays a determining role in RNAi and the sRNAs can interact with the 3′ UTR, 5′UTR, and CDS [54]. Given this heterogeneity, a near perfect match between a 20–22 nt miRNA and a bacterial target site could trigger RNAi. Our team recently discovered that plant miRNAs are found in the rhizosphere and that they affect microbial communities [2]. Transcriptomic analysis of the key rhizosphere bacterium *Variovorax paradoxus* [55] exposed to plant miRNAs revealed hundreds of differentially expressed genes [2]. Since, as mentioned above, many of the highly affected genes were in the COG category “amino acid metabolism and transportation”, we sought to further confirm the link between bacteria, amino acids, and plant miRNAs. We conducted three independent experiments to test the hypotheses that (i) exposing a soil microbial community to plant miRNAs in various amino acid sources will impact microbial community growth and amino acid uptake, (ii) that specific bacteria will respond to miRNAs leading to shifts in the microbial community composition and (iii) that plant miRNAs will reduce the amino acid consumption of the miRNA-responsive bacteria.

We first looked at the miRNAs in *Arabidopsis* roots treated with amino acids or ammonium nitrate. The miRNAs that responded positively to amino acids were mixed with those commonly found in the rhizosphere and used in vitro with a simplified soil community cultured with various amino acid sources. Bacteria that responded to miRNAs in some amino acid sources were then isolated and individually confronted to the miRNAs to assess their impact on growth and amino acid consumption.

## Material and methods

A more descriptive version of the methods is available in the supplementary material.

### Profiles of miRNA and the bacterial community in response to fertilizer treatments *in planta.*

#### Experimental design

*Arabidopsis thaliana* Col-0 (*n =* 5) were grown under three different nitrogen treatments that were supplied every 2–3 days: a mix of 17 L-amino acids (0.190 g of N/L), a no added nitrogen control and an inorganic nitrogen control (ammonium nitrate, 0.190 g of N/L). These controls enabled us to differentiate the influence of (i) nitrogen rich fertilizers vs. no added nitrogen and (ii) amino acids vs. an inorganic N source on the relative abundance of miRNAs and on the bacterial community. The growth chamber was set to 18 h of daylight and 6 h of darkness. After 21 days, the roots, rhizosphere, and distant soil were sampled and flash-frozen in liquid nitrogen.

#### miRNA profiling

The miRNA profiles were obtained by small RNA sequencing on RNA extracted from roots (RNeasy Plant Mini, Qiagen) (Illumina HiSeq4000, Centre d’expertise et de services de Génome Québec, Montreal, Canada). To associate small RNA reads to specific miRNAs [2, 56], the reads were preprocessed and trimmed [57], filtered for quality and common contaminants were removed (bbduk). These reads were mapped against a reference genome: *A. thaliana* (TAIR10/GCA_000001735.1). Alignment was carried out using the BWA parameters aln mismatch = 1 and seed = 5. Finally, the potential miRNAs that mapped against the genome were compared (BLASTn) to the miRBase hairpin and miRBase mature databases. To accurately identify the plant miRNAs induced by our fertilizer treatments, we removed all miRNAs that had been identified in our unplanted soil controls. The raw data and analyses are respectively available under NCBI BioProject accession PRJNA1107220 and on GitHub (https://github.com/le-labo-yergeau/Dozois_AA_miRNAs/tree/main/miRNA_statistics).

#### Bacterial community profiling

Since plant-microbe interactions are central to our hypotheses, we characterized the bacterial community of the *in planta* experiment via amplicon sequencing of the V4-V5 region of the 16S rRNA gene (515F-Y and 926R [58]) on a MiSeq apparatus (Illumina) at the National Research Council of Canada, (Montreal, Canada) from RNA extracted from roots. For microbial taxonomic labelling, we treated amplicon sequencing data with the pipeline AmpliconTagger [59]. This pipeline grouped the sequences into *amplicon sequence variants* (ASVs) (100% identical sequences) [60] and identified their taxonomic identity with RDP classifier using the SILVA R138 database [61–63]. The data can be found under NCBI BioProject accession PRJNA1248534 or on GitHub (https://github.com/le-labo-yergeau/Dozois_AA_miRNAs/tree/main/Microbial_statistics/In_plantae_16S).

#### Correlations and linear models linking miRNAs and bacteria

To link given taxa and our miRNAs of interest, Spearman correlations and linear regression models were performed on their relative abundances in the roots of *A. thaliana*. Spearman correlations were visualized in a heatmap involving 34 ASVs and 38 plant miRNAs. To reduce the chance of random or unreliable correlations, we kept only the ASVs that showed significant associations with at least three miRNAs. We tested the assumptions of linearity, homoscedasticity, and normality before running the regression models. Our full analysis is available on Github (https://github.com/le-labo-yergeau/Dozois_AA_miRNAs/blob/main/miRNA_statistics).

### Effects of synthetic miRNAs on a microbial community grown with different amino acids

#### Soil microbial community

We developed a simplified soil community by adding 2 g of sieved agricultural soil from our experimental field (45.5416°N, 73.7173°W) in five growth media. After 28 h (200 rpm, 25°C), the cultures were filtered (30 μm), normalized to the same optical density, combined, then pelleted (4°C, 15 min, 4700 *g*). The pellets were suspended in PBS, pooled, and aliquoted into sterile cryotubes in a cryoprotective solution [64] before being stored at −80°C.

#### Microbial growth

To verify whether miRNAs interfered with microbial activity depending on the amino acid source, five were tested. The miRNAs were synthesized with the 3′ end 2′-OH methylation specific to plant miRNAs (Integrated DNA Technologies, Supplementary Table S1) [65, 66]. The effect of these five synthetic plant miRNAs (2 μM for each miRNA) on the activity of soil microbes was evaluated. As a control, scrambled miRNAs corresponding to the five plant miRNAs were used (i.e. the sequences of ribo nucleic acids were randomized). As the precise concentration of miRNAs in the rhizosphere is not known, we chose a concentration of 2 μM based on previous work on plant and animal microbial communities [2, 42]. To test if plant miRNAs modified microbial activity, soil microbes were grown in media containing a mixture of all 17 L-amino acids (15 mM) or in media containing individual amino acids (15 mM). In all media, artificial root exudates (glucose, fructose, sucrose, lactic acid, succinic acid, and citric acid) [67] served as an additional carbon source (15 mM). We added a tetrazolium dye which turns purple in the presence of active dehydrogenases and enhances optical density. The plant miRNAs and the scrambled miRNAs were inoculated once at the beginning of the incubation. The optical density (600 nm) was measured every hour with a plate reader (Tecan Infinite M1000Pro). When microbial activity was different between the miRNA treatment and scrambled control, we repeated the experiment with individual miRNAs (2 μM) to identify which of the five miRNAs was responsible for the change (*n =* 5).

#### Amino acid quantification

We quantified the effect of individual miRNA (2 μM) on the microbial consumption of four different amino acid sources: L-proline, L-lysine, glycine, and the mix of 17 AA. The microbial community was sampled at the beginning of the experiment, at the early log-phase, the mid-log phase and the stationary phase. The amino acids were quantified (in technical duplicates) using their respective standard curves in a colorimetric assay. For quantifying L-proline and L-lysine, we added our samples to a reaction mix consisting of 1% ninhydrin, 60% acetic acid, and 20% ethanol [68] and incubated the mixture at 95°C for 20 min. Whereas for glycine, the reaction mix consisted of 1% ninhydrin diluted in 80% ethanol and was incubated for 15 min at 75°C. The optical density was read at 520 nm for L-proline and glycine and 540 nm for L-lysine (Tecan Infinite M1000Pro). The mix of 17 AA was quantified with the colorimetric L-Amino Acid Assay Kit (CELL BIOLABS INC. MET-5054) as specified by the supplier, except for the standard curve which was our own 17-AA mix. The optical density measurements were converted to amino acid concentration (μM) using the standard curve (*n =* 5).

#### Bacterial community composition

We then evaluated if the change in growth was reflected in a change within the bacterial community. We sampled the community at the endpoint of the experiment (52 h) and DNA was extracted with a physical microvolume extraction [69] and purified via ethanol-NaCl precipitation. The libraries were prepared and the V4-V5 region of the 16S rRNA gene 515F-Y and 926R was sequenced [58] (Illumina MiSeq, Centre d’expertise et de services de Génome Québec, Montreal, Canada). We labelled the taxa with RDP classifier and SILVA [61–63] as previously described. We identified specific ASVs for which the relative abundance significantly changed in response to the miRNA treatment according to both the DESeq2 [70] and ANCOM-BC [71] analyses. The data can be found under NCBI BioProject accession PRJNA1111829 or on GitHub (https://github.com/le-labo-yergeau/Dozois_AA_miRNAs/tree/main/Microbial_statistics/16SvsmiRNAs).

### Isolates challenged with miRNAs

#### Isolation of strains from the simplified soil community

We isolated the bacteria by using different solid media including a Phosphate Separately autoclaved Reasoner’s 2A meant to isolate *Chryseobacterium* and *Flavobacterium* [72]. We extracted the DNA of the isolates with a physical microvolume extraction [69], amplified the V4-V5 16S rRNA gene region, purified the PCR products (QIAquick PCR Purification Kit, Qiagen) and sent the samples for Sanger sequencing (Centre d’expertise et de services de Génome Québec, Montreal, Canada) (forward primer: 515FY and reverse primer:926R [58]). Consensus sequences were generated with the BioEdit Sequence Alignment Editor and compared to the 16S rRNA sequences of our previously identified ASVs with BLASTn. The isolates that had perfect matches with the responsive ASVs were further used.

#### Isolates of interest exposed to miRNAs

We cultured three of our isolates (*Acinetobacter*, *Chryseobacterium,* and *Raoultella*) overnight in rich media, washed the cells, normalized them (DO600 = 2.00) and challenged them once to 2 μM of individual miRNAs or 10 μM of the mix as well as the corresponding scrambled controls. The isolates were grown in media, previously described, consisting of 15 mM of amino acids and 15 mM of artificial root exudates. To stay coherent with the community experiment, we added the tetrazolium dye and measured the optical density (OD600) every hour for 52 h. In view of the large number of samples we had to measure, the first (T0) measurement was taken up to 20 min after the addition of the miRNA. We repeated the experiment five times (*n =* 5) and used paired T-tests to find timepoints and growth phases where optical density and the area under the curve differed statistically between the plant and scrambled miRNA treatments.

To quantify the effect of miRNAs on the amino acid uptake of each isolate, the isolates were grown in the 17 AA mix medium and sampled at four different timepoints (*n =* 5). The amino acids were quantified using the colorimetric L-Amino Acid Assay Kit (CELL BIOLABS INC. MET-5054) as previously described. We then calculated if the isolate’s growth and amino acid use differed between the plant and scrambled miRNA treatments. Paired T-tests were used for each timepoint and for the area under the curve, provided the assumptions were met. Otherwise, the Wilcoxon signed-rank test was used.

#### Whole genome sequencing of isolates

Isolates were cultured overnight (28°C, 200 rpm) in minimal medium and DNA was extracted with QIAmp DNA Mini Kit (Qiagen) following the protocol for Gram negative bacteria. Quantity and quality of DNA were verified prior to library preparation and Nanopore sequencing (PromethION, Oxford Nanopore technologies). The reads were combined per isolate and filtered for quality with filtlong (https://github.com/rrwick/Filtlong). The assembly was performed with Flye [73] and the expected genome size for each genus. The assembled genomes were annotated with NCBI Prokaryotic Genome Annotation Pipeline (PGAP) (https://www.ncbi.nlm.nih.gov/refseq/annotation_prok/). The data is accessible under NCBI BioProject accession PRJNA1247481.

#### Putative miRNA targets within the assembled genomes

Since the functional pairing between plant miRNAs and bacterial mRNA has yet to be validated, we used four tools to predict plant miRNA bacterial targets: psRNATarget [74], miRanda [75, 76], IntaRNA [77], and BLASTn for short queries. We only considered targets that were common between at least three tools. The sequences of all 10 miRNAs (five plant sequences and five scrambled sequences) were mapped to the coding DNA sequences (CDS) plus 100 bp upstream (to include the 5′ UTR) of each isolate that are known to be involved in amino acid transport or general nitrogen regulation.

#### Co-culture of *A. thaliana* and isolates

To test the plant-microbe resource competition relationship between *A. thaliana* and each of the three isolates, a gnotobiotic assay was performed with surface sterilized seeds. On the day of the experiment, the seeds were inoculated with washed cultures of each isolate for 2 h. The optical density at 600 nm (OD600) for each isolate was previously calculated to correspond to a concentration of 10^4^ CFU/μl. The negative control culture medium, which was washed alongside the cultures, was used to inoculate the seeds. Ten seeds were then placed in a sterile Magenta Box containing half-strength MS (Murashige and Skoog) medium with 0.8% agar. The growth chamber settings were the same as in the previous *Arabidopsis* growth experiment. The experiment lasted for 23 days. We then determined if isolates impacted *Arabidopsis* growth by comparing different plant traits with Kruskal-Wallis tests for each timepoint and for the area under the curve (*n =* 5).

## Results

### Root miRNAs shift in response to amino acids

**Figure 1:**
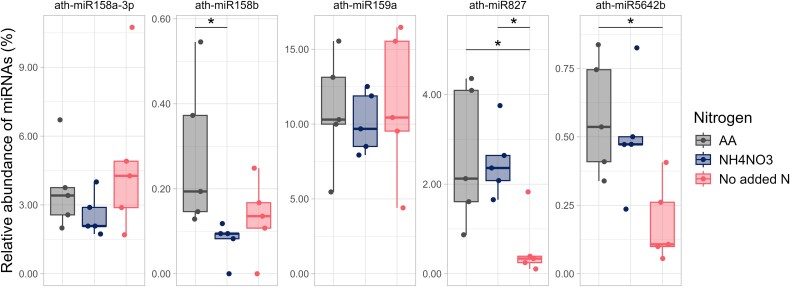
The relative abundance of the five selected miRNAs inside the roots of *A. thaliana* depending on the nitrogen treatment (AA: a mix of amino acids, NH_4_NO_3_: Ammonium nitrate and No added N). Differences in relative abundance of miRNAs between N treatments are indicated with brackets (*P*- adjusted with Holm correction <.05, *n =* 5).

To identify root miRNAs that responded to nitrogen inputs, we grew *Arabidopsis* under three fertilizer treatments (a mix of 17 L-amino acids, a no added nitrogen control and an inorganic nitrogen control (NH_4_NO_3_)). Amino acids increased the relative abundance of six root miRNAs (ath-miR158b, ath-miR160a-3p, ath-miR166b-5p, ath-miR390a-3p, ath-miR827 and ath-miR5642b) (Supplementary Fig.S1). Among the 10 most abundant root miRNAs, the relative abundance of only ath-miR408 was different among N treatments (Supplementary Fig. S2): this miRNA was less abundant under amino acid fertilization than the NH_4_NO_3_ treatment. Five miRNA candidates were selected for further experiments: miRNAs that positively responded to the amino acid treatment (ath-miR158b, ath-miR827 and ath-miR5642b) and miRNAs abundant in the rhizosphere that were shown to shift the amino acid gene expression in *V. paradoxus* (ath-miR158a-3p and ath-miR159a) [2] (Fig. 1).

### Bacterial ASVs correlate to the relative abundance of miRNAs

To highlight links between the relative abundance of certain taxa and the relative abundance of miRNAs, in the roots of *Arabidopsis* treated with three N treatments, we performed Spearman correlations and compiled those that were significant (*P* < .05) (Supplementary Table S2). Although there were no correlations for ath-miR158b, three were significant for ath-miR158a-3p, one for ath-miR159a, five for ath-miR827 and three for ath-miR5642b (Supplementary Fig. S3). Half of the correlations were positive, and the others were negative. Among the taxa correlated to miRNA, some also responded to our N treatments (Supplementary Fig. S4). Five of our correlations involved N-responding miRNAs and N-responding taxa, suggesting that indirect miRNA-microbe interactions were more likely at play. With linear regression models, we confirmed that the N treatments were also important in influencing the abundance of *Massilia* (ASV#14) and *Niastella* (ASV#67) (Supplementary Table S3). When the variable “N treatments” was partialled out from our linear regression models, 7/12 of the ASV-miRNA pairs were still significant (*P <* .05, Table 1). The models that were more suggestive of direct miRNA-microbe interactions included: Ktedonobacteria (ASV#111) ~ ath-miR158a-3p, Ktedonobacteria (ASV#111) ~ ath-miR827 and *Litorilituus* (ASV#630) ~ ath-miR158a-3p.

**Table 1:** Linear regressions between the relative abundances of miRNA and bacterial taxa.

	ASV	miRNA	Adjusted R^2^	*P*-value
N-responding ASVs correlated to N-responding miRNAs	#14 *Massilia*	ath-miR827 ath-miR5642b	0.56 0.26	.00075^***^ .030^*^
	#41 *Luteimonas*	ath-miR827	0.27	.027^*^
Non N-responding ASVs correlated to miRNAs	#111 Ktedonobacteria	ath-miR158a-3p ath-miR827	**0.31** 0.31	.019^*^ .019^*^
	#630 *Litorilituus*	ath-miR158a-3p ath-miR827	**0.22** 0.21	.043^*^ .048^*^

Linearity, normality and homoscedasticity assumptions were validated. Negative relationships are identified in **bold** (*n =* 5).

### Plant miRNAs impact the growth of soil microbes and the amino acid use of bacterial isolates causing community shifts

Growth and amino acids measurements were performed on true biological replicates in separated experiments, which varied considerably. Statistical tests were performed using paired samples from the same experiments. Accordingly, differences observed in the Figures and standard deviation across all samples might not reflect the results of the paired statistical tests.

**Figure 2:**
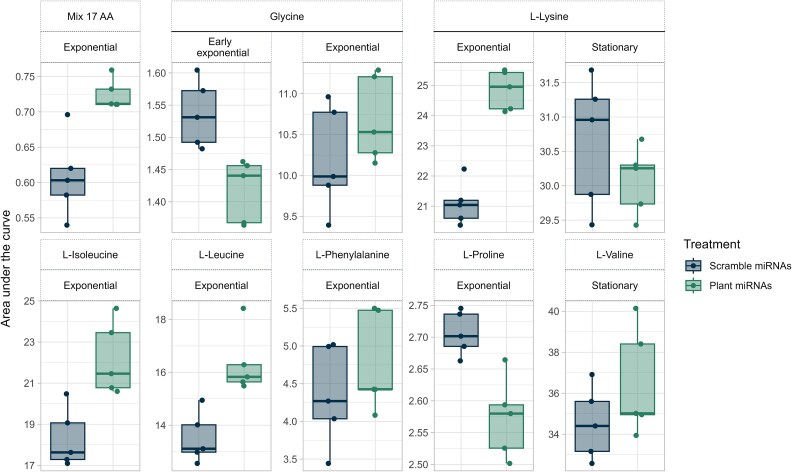
The miRNA treatments impact the area under the growth curve during key growth phases. The simplified soil community’s growth phases were different (*P <* .05, paired T-test, *n =* 5) between the miRNA treatments (10 μM of mix of plant miRNAs vs. 10 μM of a mix of scrambled miRNAs) in these eight amino acid sources.

**Figure 3:**
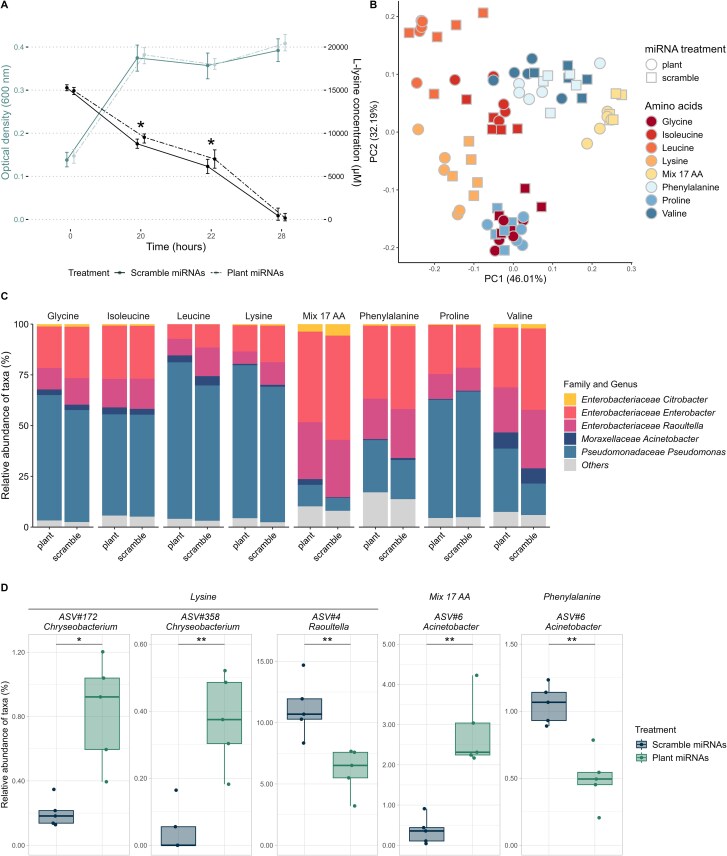
miRNAs modify bacterial composition and L-lysine consumption. (A) Microbial growth (OD600, blue lines) and L-lysine consumption (black lines) over time (hours). Dashed lines indicate that the microbes were exposed to 2 μM of ath-miR159a whereas full lines indicate that the microbes were exposed to 2 μM of scrambled ath-miR159a. Significant results are identified with a ^*^ (*P <* .05 paired T-test, *n =* 5). Error bars are 95% confidence intervals. (B) Principal component analysis of bacteria grown in different amino acid sources and exposed to the mix of miRNAs (10 μM) (Hellinger transformation, *n =* 5). (C) Composition of the soil microbial community grown with different amino acids as a nitrogen source (mean relative abundance, *n =* 5). (D) ASVs for which the relative abundance was different (*P <* .05) between the miRNA treatments (10 μM of mix of plant miRNAs vs. 10 μM of a mix of scrambled miRNAs). The relative abundance of the ASVs was found to be significantly different for both DESeq2 and ANCOM-BC analyses (*n =* 5).

We first synthesized the five plant miRNAs and scrambled controls (Fig. 1 and Supplementary Table S1) to evaluate if they interfered with microbial growth across different amino acid sources using a tetrazolium dye as a proxy for microbial activity. The mixture of miRNAs increased or decreased the growth of the community for at least two consecutive timepoints (2 h) for 13 out of the 18 amino acid sources (Table 2 and Supplementary Fig. S5) and differences in the area under the growth curve were observed for eight of the 18 amino acid sources (Fig. 2 and Supplementary Table S4). For most of the amino acid sources, the effect of miRNAs on microbial growth occurred during the exponential phase (Fig. 2 and Supplementary Table S4). To assess miRNA effects on community growth, two nitrogen sources were tested for negative impacts (L-proline, glycine) and two for positive impacts (17 L-amino acid mix, L-lysine). For L-proline and glycine, ath-miR827 and ath-miR5642b reproduced the negative effect (Supplementary Fig. S6, Supplementary Table S5). For the mix of 17 L-AA and L-lysine both ath-miR159a and ath-miR827 had positive effects. Ath-miR158a-3p only positively affected microbial growth in L-lysine (Supplementary Fig. S6, Supplementary Table S5). However, the effects that lasted throughout the exponential growth phase were all positive (Supplementary Table S5). The bacterial community responded more strongly to miRNAs when cultured in L-lysine (responded to 3/5 miRNAs) where ath-miR159a produced the most lasting effects (Supplementary Table S5). For each of the miRNA-AA source pairs found in Supplementary Fig. S6, the effect of miRNAs on microbial AA consumption was also tested (Supplementary Fig. S7). Microbes reduced their consumption of L-lysine when exposed to ath-miR159a (2 μM) compared to its scrambled control (Fig. 3A) whereas miRNAs did not impact the uptake of any other amino acids.

**Table 2:** Effect of 10 μM of miRNAs (plant miRNAs compared to scrambled miRNAs) on the activity (OD600) of soil microbes.

Effect on microbial activity	Amino acids
Positive effect	L-asparagine L-alanine L-aspartic acid L-isoleucine L-leucine L-lysine HCl^***^ L-phenylalanine L-valine Mix of all 17 AA
Negative effect	L-proline L-glutamic acid^**^ Glycine^***^ L-tryptophan
No effect^*^	L-arginine HCl L-cysteine HCl L-histidine L-methionine L-serine

^*^Or only one timepoint presented significant effects.

^**^Significant effects for only two inconsecutive timepoints.

^***^The effect changed later in the growth phase. (Paired T-tests, *n =* 5).

Since this was the only case where we observed a direct effect on amino acid consumption, we hypothesized that the effects on microbial growth must stem from shifts in community composition. Taxonomic bacterial changes caused by miRNAs were investigated for eight amino acids (glycine, L-isoleucine, L-leucine, L-lysine, L-phenylalanine, L-proline, L-valine, Mix of 17 AA) by 16S rRNA gene sequencing (Fig. 3B and C). Our medium, composed of 17 amino acids, favored bacteria of the family Enterobacteriaceae compared to media containing one type of amino acid. The bacterial community exposed to a single miRNA was mainly influenced by the type of amino acid provided (R^2^ = 0.1427, *P* = .001, Supplementary Table S6, Supplementary Fig. S8) whereas the bacterial community exposed to a mix of miRNAs was influenced by the interaction of the miRNA treatment (plant vs. scrambled) and the type of amino acid provided (R^2^ = 0.1478, *P =* .013, Supplementary Table S6).

Microbial community changes, such as a lower abundance of *Raoultella* and *Enterobacter,* were induced by the mix of plant miRNAs at the family and genus level for most nitrogen sources (*P <* .05)*.* Interestingly, L-isoleucine instigated no obvious microbial changes at this taxonomic level (Fig. 3C). At the ASV level, we identified four ASVs differentially abundant between the miRNA treatments (plant vs. scrambled) (*P <* .05): ASV#4-*Raoultella,* ASV#6-*Acinetobacter*, ASV#172-*Chryseobacterium* and ASV#358-*Chryseobacterium* (Fig. 3D). Plant miRNAs negatively impacted the relative abundance of *Raoultella* (ASV#4) grown in L-lysine. The other taxa *Chryseobacterium* (ASV#172 and ASV#358) cultured in L-lysine were positively affected by plant miRNAs. A particularity occurred with ASV#6-*Acinetobacter* which seemed to prosper when exposed to plant miRNAs in a mix of amino acids but exhibited the opposite phenotype when cultured in L-phenylalanine.

**Figure 4:**
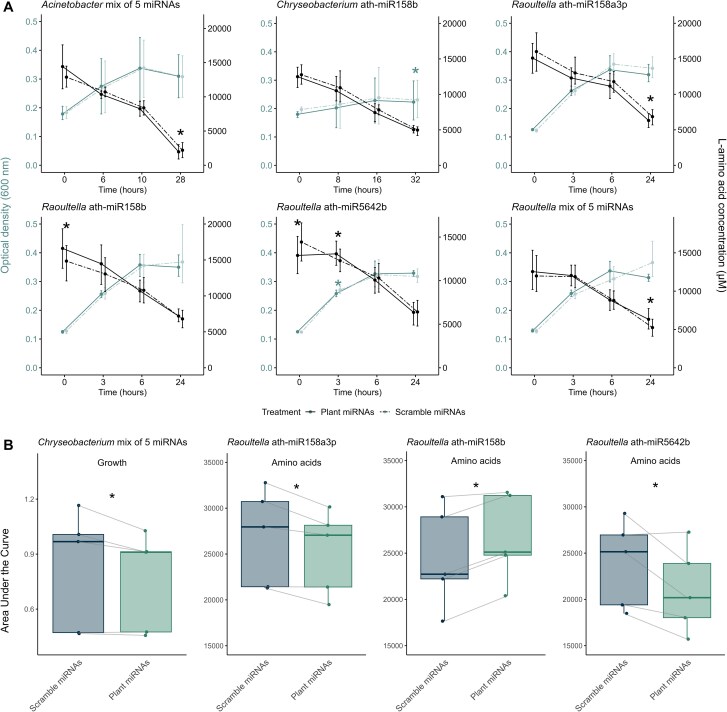
The miRNA treatments affect how isolates grow and the rate at which they uptake amino acids from the 17 AA mix media. (A) Microbial growth (OD600, blue lines) and amino acid consumption (black lines) over time (hours). Full lines indicate that the microbes were exposed to plant miRNAs whereas dashed lines indicate that the microbes were exposed to scrambled miRNAs. Significant results are identified with a ^*^ (*P <* .05 paired T-test, *n =* 5). Error bars are 95% confidence intervals. (B) Differences in the area under the curve for *Chryseobacterium* growth and for *Raoultella* amino acid uptake (*P <* .05 paired T-test, *n =* 5).

We isolated three strains of miRNA-responding ASVs that perfectly matched the 16S rRNA gene sequences identified in the community data: ASV#4-*Raoultella,* ASV#6-*Acinetobacter*, ASV#172-*Chryseobacterium*. We cultured each isolate with the mix of miRNAs and individual miRNAs. The largest changes in isolate growth were caused by ath-miR158b (Supplementary Tables S7 and S8). The positive effects of plant miRNAs on the growth of *Acinetobacter* cultured in the AA mix were coherent with the effects at the community level (Fig. 3D). The amino acid uptake of *Acinetobacter* was 14.8 ± 10.4% (average ± standard deviation) higher in response to the mix of plant miRNAs at the endpoint of the experiment (amino acid concentration left in the growth media of 1961 ± 1395 μM for plant miRNAs vs 2195 ± 1431 μM for scramble miRNAs, Fig. 4A). When *Acinetobacte*r was grown in L-phenylalanine, the mix of miRNAs only positively impacted the growth which was unexpected, though 48 h postinoculation some of the replicates treated with the scrambled control had a growth spurt (Supplementary Fig. S9). Whereas the five plant miRNAs positively impacted the relative abundance of *Chryseobacterium* in the soil community, in pure culture ath-miR158b caused the opposite effect, with a 3.9 ± 3.8% growth reduction (OD600 0.22 ± 0.08 for plant miRNA vs 0.23 ± 0.08 for scramble, Fig. 4A, Supplementary Tables S7 and S8 and Supplementary Fig. S9). The prolonged negative effect of ath-miR158b on the growth of *Raoultella*, may also explain its negative response to plant miRNAs within the simplified microbial community. *Raoultella* was the only isolate both negatively impacted by the mix of five plant miRNAs in the soil community and in pure culture. The mix of five miRNAs and the plant miRNA ath-miR158b reduced the amino acid uptake of *Raoultella* by 20.7 ± 7.8% (6324 ± 1750 μM for plant miRNAs vs. 5247 ± 1474 μM for the scramble miRNAs) and by 11.8 ± 2.8% (16 607 ± 3508 μM for plant miRNAs vs. 14 841 ± 3029 μM for the scramble miRNAs), respectively (Fig. 4A and B). Yet, we found that plant miRNAs ath-miR158a-3p and ath-miR5642b could stimulate higher amino acid uptake within a 24-h period (Fig. 4B). Curiously, ath-miR5642b initially stimulated amino acid uptake by 10.5 ± 9.0% (12 894 ± 2929 μM for plant miRNAs vs. 14 431 ± 2970 μM for the scramble miRNAs) at the start of the experiment, but 3 h later, it delayed amino acid uptake by 6.6 ± 5.2% (13 078 ± 1816 μM for plant miRNAs vs. 12 288 ± 1741 μM for the scramble miRNAs) and growth by 4.7 ± 4.2% (OD 600 of 0.258 ± 0.01 for plant miRNAs vs. 0.271 ± 0.02 for the scramble miRNAs) compared to the scrambled control. Whole genome sequencing revealed that, among the three isolates, *Raoultella* had the most diverse set of inorganic nitrogen and amino acid transporters (Supplementary Table S9) suggesting its potential to mitigate the negative effects caused by a specific miRNA.

### miRNAs ath-miR159a and ath-miR827 potentially target more amino acid transportation genes

To identify potential N-related gene targets in the genomes of our three isolates, we performed sRNA-mRNA interaction predictions. Our target predictions identified a total of 12 plant miRNA targets and six scrambled miRNA targets (Supplementary Table S10). A total of seven predicted targets were identified for *Acinetobacter*, one for *Chryseobacterium* and 10 targets for *Raoultella*. This was consistent with the number of CDS related to amino acid transportation and nitrogen regulation we identified for each isolate: 19, 4, and 67 CDS for *Acinetobacter, Chryseobacterium,* and *Raoultella,* respectively. For *Acinetobacter*, ath-miR159a was predicted to target the most genes including a sodium/proline symporter, a serine/threonine transporter and a *glnG* nitrogen regulation protein NR(I) while its scrambled equivalent was predicted to target none. For *Raoultella,* ath-miR827 was predicted to target the most genes with four different targets including: a an *abgT* p-aminobenzoyl-glutamate transporter and three amino acid ABC transporters permeases/ATP-binding proteins. No predicted targets were identified for the scrambled version of ath-miR827. For *Raoultella,* ath-miR5642b was also predicted to target three different genes: a glycine betaine/L-proline transporter, an aromatic amino acid DMT transporter and a tryptophan permease whereas its scrambled version was predicted to target none. We only found two hits in the 100 bp upstream (including the 5′UTR region) of the CDS. Both hits were for scrambled miRNA (scramble-ath-miR158a-3p and scramble-ath-miR159a) in *Raoutella*’s genome and were more than 50 bp upstream of the coding region. Overall, the miRNAs that were predicted to target the most genes related to amino acid transportation were ath-miR159a, for *Acinetobacter*, and ath-miR827 for *Raoultella*. These miRNAs were also found to have the greatest impact on the soil community’s amino acid use and growth, respectively.

### The isolate *Raoultella* delayed the germination of *Arabidopsis* and in time killed the plants

**Figure 5:**
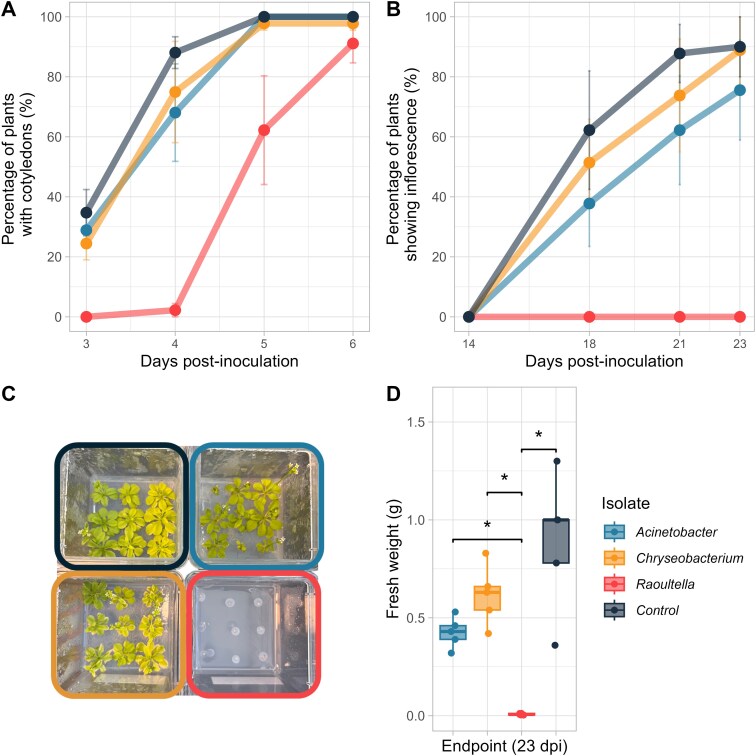
Effect of the three isolates (*Acinetobacter*: light blue, *Chryseobacterium*: yellow, *Raoultella*: red, negative control: dark blue) on *Arabidopsis* germination and development. (A) Percentage of plants with cotyledons, (B) percentage of plants showing inflorescence (floral stem), (C) picture of plants 23 days postinoculation and D. Fresh weight of the plants 23 days postinoculation. (Wilcoxon–Mann–Whitney test, *P* adjusted with Benjamini–Hochberg correction, *n =* 5). Error bars in A and B are standard error.

We hypothesized that the positive or negative effect of miRNAs on the isolates would correlate with their beneficial or detrimental effect on plant growth. We inoculated our three isolates on surface-sterilized *Arabidopsis* seeds and followed germination and growth. The plants inoculated with *Raoultella* germinated two days later than the rest of the plants (Fig. 5A). Even though they germinated, all the plants inoculated with *Raoultella* died mid-experiment. None of these plants reached flowering (Fig. 5B), and by day 23, when we took a picture and weighted the plants, they were all dead (Fig. 5C and D). The *Acinetobacter* and *Chryseobacterium* isolates did not significantly affect *Arabidopsis* (Fig. 5).

## Discussion

We had previously shown that *V. paradoxus*—a key rhizosphere bacterium—shifted the expression of many genes in the “amino acid transport and metabolism” COG category in response to exposure to plant miRNAs [2]. Here we showed that (i) amino acid fertilization changed the relative abundance of miRNAs in *Arabidopsis* roots; (ii) as hypothesized these miRNAs affected a simplified soil bacterial community, where ath-miR827 most effectively altered soil bacterial growth, (iii) miRNAs elicited the strongest bacterial response when they were grown in L-lysine, and (iv) plant miRNAs impacted the growth and amino acid use of certain isolates from the simplified soil bacterial community. As expected, we detected miRNA-responsive bacteria, affected in terms of growth and amino acid uptake, likely explaining the strong shifts observed within the soil community. However, the predominantly positive effects of plant miRNAs were unexpected. Our results are suggesting that, depending on the availability of nutrients, plant use miRNA to fine-tune their rhizospheric microbiota, inhibiting competitive or pathogenic bacteria and facilitating neutral or beneficial bacteria.

We identified a dozen of miRNAs in the roots of *A. thaliana* that responded to nitrogen, many of which were among the most abundant miRNAs in the rhizosphere [2]. Within the plant, specific miRNAs regulate N metabolism and respond to different exogenous N treatments [29–33]. For instance, ath-miR827 is known to contribute to phosphate homeostasis in a nitrate-dependant manner [32, 78–80]. Like previous research [32, 79], we also found ath-miR827 to be less abundant in the roots of *A. thaliana* in low N conditions. We found ath-miR827 to be the most correlated to bacterial taxa in plant roots and it was retained in four out of seven of our regression models. However, in this *in planta* experiment, it was challenging to separate the direct effects of amino acids on the bacterial community from the indirect effects due to shifts in plant miRNAs. Indeed, amino acids can influence microbe-microbe interactions, community composition, and functional diversity by serving as carbon and nitrogen sources and precursors for bioactive molecules that promote plant growth and facilitate microbe signaling [81, 82]. Some amino acids, like L-tryptophan, can enhance bacterial functions, while others, such as L-methionine, L-valine, L-cysteine, and L-serine, can inhibit growth and IAA production [83]. Amino acids like L-phenylalanine can drive stronger bacterial shifts through bioactive molecule production or selective pressure [84]. To distinguish direct from indirect effects, we conducted in vitro experiments with a simplified soil bacterial community. We chose to test N-responding miRNAs (ath-miR158b, ath-miR827 and ath-miR5642b) and miRNAs known to be abundant in the rhizosphere (ath-miR158a-3p and ath-miR159a).

As previously reported, we showed here that bacterial community and isolated strains can respond differently—even oppositely—to different plant miRNAs. The first case is when a single miRNA has a different effect on different bacteria. Previously, bol-miR159 triggered taxa-dependent negative (*Bacillus*) or positive (*Weissella* and *Ralstonia*) effects [43]. In another study, among four to five human miRNAs predicted to target two gut bacteria, only one was shown to promote the growth of *Fusobacterium nucleatum* (hsa-miR-515-5p) and one to promote the growth of *Escherichia coli* (hsa-miR-1226-5p) suggesting again that miRNAs differentially affect bacteria [42]. In a mixed community assessed using methods that produce proportional data—such as 16S rRNA gene sequencing—, this positive effect could be due to an inhibition of a community member that would result in an apparent increase in other community members. To clarify this, we also isolated three of the most responsive community members and confirmed that they also responded in isolation, both positively and negatively. During stationary growth, the mixture of five plant miRNAs promoted amino acid consumption by *Acinetobacter* while reducing it in *Raoultella*. All three isolates showed varying growth responses to plant miRNAs: *Acinetobacter* grew more, *Raoultella* grew less, and *Chryseobacterium* was affected either way depending on the miRNA. This suggests that—in contrast to our hypothesis—the effect of plant miRNAs extends beyond competitive interactions and may be involved in the enrichment of specific bacteria. Although most reports of sRNAs exchange involve a plant host and a pathogen, *Rhizobium* can deliver sRNA fragments to soybean cells to control nodule initiation and development [50]. Arbuscular mycorrhizal fungi, another key symbiont, have also been shown to exchange sRNAs with the plant to benefit their mutualistic interactions [49, 85, 86]. It appears that a miRNA could be used by the plant to fine-tune the microbial community composition, by either inhibiting or enhancing the activities of different members of the microbiota.

The second case is when different miRNAs have different effects on the same bacterium. For instance, the growth of *Chryseobacterium* in the mixture of amino acids was negatively impacted by ath-miR158b and ath-miR827, and positively by ath-miR5642b, whereas the growth of *Raoultella* in L-Lysine was positively affected by ath-miR159a and negatively by ath-miR158a-3p, ath-miR158b, and ath-miR5642b. Similarly, different plant miRNAs were shown to induce different responses in growth and activity of *Lactobacillus*, meaning that a combination of miRNAs is unlikely to have a synergetic effect [37]. Here, the community composition of our simplified soil bacterial community responded more to a combination of miRNAs than to single miRNAs. At a community level, a diverse mixture of miRNAs interacting with a range of bacterial targets would more likely result in a detectable effect. The variety of miRNAs likely interferes with different pathways within various bacterial species, leading to stronger responses. Here again, this suggests that the plant could use subtle variations in the miRNA content of its rhizosphere to fine-tune the microbial community composition.

On top of these miRNA- and taxa-specific responses of the bacterial community, the responses were shown here to vary with amino acids. For instance, the soil community growth was both positively (mix of 17AA and L-lysine) and negatively (glycine and L-proline) affected by ath-miR827. This miRNA has also been shown to limit the entry of *L. rhamnosus* into gut epithelial cells [37], but we show here that its effect is modulated by nutrients. Similarly, nutrients have been shown to modulate DNA uptake: transformation of *P. stutzeri* was more frequent at the onset of the stationary phase under nutrient limitation [87], while for *A. calcoaceticus*, a nutrient upshift with phosphate salts in the soil microcosms enhanced transformation rates [88]. Also, microbes cultured with L-lysine were more responsive to plant miRNAs. L-lysine has been shown to increase cell permeability or cell wall damage of bacteria such as *A. baumannii*, *E. coli*, *Klebsiella pneumoniae*, making them more susceptible to antibiotics [89, 90]. Perhaps bacteria grown in L-lysine were also more likely to take up miRNAs because of an increased cell permeability. Lysine is a particularly interesting amino acid, as it may be protected from soil microbial decay because of its positive charge which allows it to bind to negatively charged soil particles [91, 92]. Plants have Lysine-Histidine Transporters (*LHTs*) [93, 94] and Amino Acid Permeases (*APPs*) [95, 96] that can uptake L-lysine from the soil making them good competitors for this slowly degrading N source. Plants can use *LHTs* to uptake more amino acids as a response to microbe-associated molecular patterns (MAMPs) and consequently reduce microbial growth [97]. Although L-lysine is found in root exudates [98, 99], the increased leaching of lysine could benefit pathogens, as L-lysine is a key precursor for plant systemic acquired resistance (SAR) to infections [100]. L-lysine has also been shown to serve as a nutrient source for the soil bacterium *P. putida* [101, 102], suggesting that it also helps microbes colonize the rhizosphere.

Since we showed that *Arabidopsis* modulates the miRNA composition of its root environment, depending on the amount and type of nitrogen available, we could also envision indirect—through miRNAs—effects of nutrient availability on the bacterial community, on top of the direct effects discussed above. In fact, in our *in planta* experiment we found some significant relationships between individual bacteria and plant miRNAs even when partialling out the effect of fertilization. This suggests that, through a nutrient-induced modulation of root miRNAs, plants could influence the microbial community composition.

Among our microbial community, *Raoultella* appeared as the most affected by plant miRNAs. *Raoultella* was indeed the only isolate, both negatively impacted by the mix of five plant miRNAs in the simplified soil community and in pure culture. We hypothesized that *Raoultella* would be detrimental for plant growth and confirmed that it negatively impacted *Arabidopsis* germination and growth. Our isolate also had numerous amino acid transporters encoded in its genome, which is typical for highly competitive bacteria and/or pathogens [103–105]. Consequently, the plant miRNAs used were predicted to target more genes in the *Raoultella* genome as compared to the other isolates. This overrepresentation of target genes might explain why we found more coherent effects of the various miRNAs under different conditions for *Raoultella*, as compared to *Chryseobacterium* and *Acinetobacter*. These two latter bacteria were sometimes favored and sometimes inhibited by plant miRNAs, had lower amounts of N-related genes in their genomes—and consequently harbored less predicted targets—, and finally did not have detrimental effects on *Arabidopsis*. We speculate that the increased presence of N-related genes—a hallmark of pathogens and efficient competitors for N—could suffice for these bacteria to be more targeted by plant miRNAs.

Here we showed that plant miRNAs modify bacterial growth, relative abundance, and amino acid consumption, depending on the amino acid source supplemented. Taken together our results suggest that plants use miRNAs to fine tune the microbial community depending on the soil nutrient status. Having miRNAs that can increase or decrease various community members depending on their competitive or cooperative nature under nutrient limitation or not would be immensely advantageous for plants. It also suggests an avenue to use miRNAs to modify plant-associated microbial communities, in response to nutrient status, towards improving agricultural sustainability.

## Supplementary Material

Supplementary_Tables_Figures_ycaf206

Supplementary_Methods_ycaf206

## Data Availability

The sequencing data generated in this study has been deposited under NCBI BioProject accessions PRJNA1107220 (root miRNAs), PRJNA1248534 (root, rhizosphere and bulk soil 16S rRNA gene amplicons), PRJNA1111829 (simplified soil community 16S rRNA gene amplicons) and PRJNA1247481 (whole genome sequence of isolates). The R code used to analyse the data and generate the figures is available on GitHub (https://github.com/le-labo-yergeau/Dozois_AA_miRNAs).
