# Supplementary material for: Plant miRNAs influence soil bacterial growth and amino acid uptake, restructuring community composition
Source: ISME Commun. 2025 Nov 8;5(1):ycaf206. doi: 10.1093/ismeco/ycaf206 (PMC12648395; doi:10.1093/ismeco/ycaf206)
Supplement: Supplementary_Tables_Figures_ycaf206 [file supplementary_tables_figures_ycaf206.docx]

**Supplementary material for:**

**Plant miRNAs influence soil bacterial growth and amino acid uptake, restructuring community composition.**

Jessica A. Dozois^1^, Marc-Antoine Duchesne^1^, Katel Hallaf^1^, Julien Tremblay^1^ and Étienne Yergeau^1*^

^1^ Institut National de la Recherche Scientifique, Centre Armand-Frappier Santé Biotechnologie, Laval, Québec, Canada.

*Corresponding author: [Etienne.Yergeau@inrs.ca](mailto:Etienne.Yergeau@inrs.ca)

Table S1: Sequences of the single-stranded synthetic miRNAs.

| **miRNA** | **Sequences (5’ to 3’)** |
| --- | --- |
| ath-miR158a-3p | UCCCAAAUGUAGACAAAGCA |
| ath-miR158b | CCCCAAAUGUAGACAAAGCA |
| ath-miR159a | UUUGGAUUGAAGGGAGCUCUA |
| ath-miR827 | UUAGAUGACCAUCAACAAACU |
| ath-miR5642b | UCUCGCGCUUGUACGGCUUU |
| sc-ath-miR158a-3p | UGAAAACAUACAUCAGACCG |
| sc-ath-miR158b | GACAAGCUCCACAUACAGAA |
| sc-ath-miR159a | AGAGAGACCUUGGGUUGAUUU |
| sc-ath-miR827 | GAUCCAAAAAGCUAAUUUCAC |
| sc-ath-miR5642b | ACGUCGUAAAACUUCACGUG  UCCGGGCUUUGUACCUCUUG* |

All synthesized miRNA sequences have a 2’-OH methylation at the 3’ end. *Used in the isolates vs. miRNA experiment only.

Table S2: Summary of Spearman correlations between the relative abundances of root miRNAs and bacterial taxa.

|  | | **ASV** | **miRNA** | **R_S_ *p*-value** |
| --- | --- | --- | --- | --- |
| N-responding ASVs correlated to N-responding miRNAs | #14 *Massilia* | | ath-miR827  ath-miR5642b | 0.73 0.0029 *  0.60 0.020 * |
|  | #41 *Luteimonas* | | ath-miR827 | 0.70 0.0052 * |
|  | #478 *Massilia* | | ath-miR827  ath-miR5642b | **-0.72** 0.0036 *  **-0.53** 0.044 * |
| Non N-responding ASVs correlated to miRNAs | #53 *Flavitalea* | | ath-miR159a | 0.55 0.036 * |
|  | #67 *Niastella* | | ath-miR158a-3p | **-0.63** 0.014 * |
|  | # 98 *Rhizobiaceae* | | ath-miR5642b | **-0.57** 0.030 * |
|  | #111 *Ktedonobacteria* | | ath-miR158a-3p  ath-miR827 | **-0.55** 0.038 *  0.61 0.018 * |
|  | #630 *Litorilituus* | | ath-miR158a-3p  ath-miR827 | **-0.52** 0.047 *  0.64 0.013 * |

Table S3: Linear regressions of the relative abundance of bacterial taxa explained by the relative abundance of root miRNAs and N treatments.

| **ASV** | **miRNA** | **Adjusted R^2^** | **F-statistic** | ***p*-value** | **Significant coefficients** | **Relationship** |
| --- | --- | --- | --- | --- | --- | --- |
| #14 *Massilia* | ath-miR827  ath-miR5642b | 0.6263  0.4793 | 8.822  5.295 | 0.002892 *  0.01672 * | ns  Nitrogen | positive  positive |
| #67  *Niastella* | ath-miR158a-3p | 0.4002 | 4.113 | 0.03489 * | miRNA | negative |

Linearity, normality and homoscedasticity assumptions were validated.

Table S4: Specific growth phases affected by the mix of plant miRNAs

| **N Source** | **Growth phase affected (hours)** | ***p*-value** | **Effect of plant miRNAs** |
| --- | --- | --- | --- |
| Glycine | Early Exponential (15-18)  Exponential (19-27) | 0.004987  0.005308 | Negative  Positive |
| L-isoleucine | Exponential (39-52) | 0.003337 | Positive |
| L-leucine | Exponential (39-52) | 0.0002904 | Positive |
| L-lysine | Exponential (23-38)  Stationary (39-52) | 0.0001937  0.02804 | Positive  Negative |
| L-phenylalanine | Exponential (20-33) | 0.002841 | Positive |
| L-proline | Exponential (0-16) | 0.002502 | Negative |
| L-valine | Stationary (22-52) | 0.007254 | Positive |
| Mix of 17 L-AA | Exponential (0-5) | 0.006503 | Positive |

Paired T-tests of the area under the curve of each growth phase were performed.

Table S5: Growth phases affected by individual miRNAs (2 µM).

| **N Source** | **miRNA** | **Growth phase affected (hours)** | ***p*-value** | **Effect of plant miRNAs** |
| --- | --- | --- | --- | --- |
| glycine | ath-miR827 | None | N.A. | N.A. |
|  | ath-miR5642b | None | N.A. | N.A. |
| L-lysine | ath-miR158a-3p | Exponential (25-37) | 0.03404 | Positive |
|  | ath-miR159a | Exponential (25-37)  Stationary (38-40) | 0.01544  0.03895 | Positive |
|  | ath-miR827 | Exponential (25-37) | 0.03032 | Positive |
| L-proline | ath-miR827 | None | N.A. | N.A. |
|  | ath-miR5642b | None | N.A. | N.A. |
| Mix of 17 L-AA | ath-miR159a | Exponential (4-28) | 0.007735 | Positive |
|  | ath-miR827 | Exponential (4-28) | 0.02033 | Positive |

Paired T-tests of the area under the curve of each growth phase were performed.

Table S6: Permutational multivariate analysis of variance of the bacterial communities (16S rRNA gene) exposed to miRNAs and cultured in media with different amino acids.

|  |  | **Df** | **SumOf**  **Sqs** | **R^2^** | **F** | **Pr(>F)** |
| --- | --- | --- | --- | --- | --- | --- |
| Exposed to the mix of 5 miRNAs | **Amino acids** | 7 | 1.0225 | 0.0857 | 1.0911 | 0.329 |
|  | **miRNA treatment** | 1 | 0.5729 | 0.0480 | 4.2793 | **0.011*** |
|  | **Amino acids: miRNA treatment** | 7 | 1.7629 | 0.1478 | 1.881 | **0.013*** |
|  | Residual | 64 | 8.5676 | 0.7184 |  |  |
|  | Total | 79 | 11.9259 | 1 |  |  |
| Exposed to single miRNAs | **miRNA treatment** | 1 | 0.01848 | 0.002015 | 0.2056 | 0.894 |
|  | **miRNA family** | 3 | 0.256569 | 0.027973 | 0.9515 | 0.444 |
|  | **Amino acids** | 3 | 1.308915 | 0.142705 | 4.8541 | **0.001*** |
|  | **miRNA treatment: miRNA family** | 3 | 0.471203 | 0.051373 | 1.7475 | 0.099 |
|  | **miRNA treatment: Amino acids** | 3 | 0.271417 | 0.029591 | 1.0065 | 0.41 |
|  | **miRNA family: Amino acids** | 2 | 0.2904 | 0.031661 | 1.6154 | 0.182 |
|  | **miRNA treatment: miRNA family: Amino acids** | 2 | 0.083521 | 0.009106 | 0.4646 | 0.805 |
|  | Residual | 72 | 6.471647 | 0.705576 |  |  |
|  | Total | 89 | 9.172152 | 1 |  |  |

Table S7: Isolates affected by miRNAs at different time points (h) of their growth phase.

| **Isolate** | **Culture media** | **miRNA** | **Effect of plant miRNA** | **Significant time points** |
| --- | --- | --- | --- | --- |
| *Raoultella* (ASV#4) | L-lysine | ath-miR158b | Negative | 3, **11-15**, **17-32**, **44-51** |
|  |  | ath-miR158a-3p | Negative | 19,23,**35-37** |
|  |  | ath-miR159a | Positive | 16,33,35 |
|  |  | ath-miR5642b | Negative | 1,5,11,23,26 |
|  | 17 L-AA mix | ath-miR158b | Negative | 7,10,**12-19** |
| *Acinetobacter* (ASV#6) | 17 L-AA mix | ath-miR158a-3p | Positive | 2 |
|  |  | ath-miR827 | Positive | 0,1,3 |
|  |  | ath-miR5642b | Positive | **6-8**,12,49,51 |
|  | L-Phe | ath-miR158b | Positive | 23, **28-39**, 45,47,**49-51** |
|  |  | ath-miR159a | Negative | 46,51 |
|  |  | ath-miR5642b | Positive | **25-29**, **31-34**, 36 |
|  |  | miRNA mix | Positive | 0, **5-7**, 9,11, 13, **15-19**, **21-26** |
| *Chryseobacterium* (ASV#172) | 17 L-AA mix | ath-miR158b | Negative | 1,3,**5-22**, **26-28**, 33,**39-41** |
|  |  | ath-miR827 | Negative | 4,5,17,19,20,21,25 |
|  |  | ath-miR5642b | Positive | 6,7 |
|  |  | miRNA mix | Positive | 16,18,45,46,51 |

The positive or negative impacts of plant miRNAs (*p* <0.05) on specific timepoints of the growth phase of cultures treated with the tetrazolium dye. Time intervals are identified in bold. Paired T-tests for each time point were performed (n=5).

Table S8: Growth phases of the isolates affected by plant miRNAs.

| **Isolates** | **Culture media** | **miRNA** | **Growth phase affected (hours)** | ***p*-value** | **Effect of plant miRNA** |
| --- | --- | --- | --- | --- | --- |
| *Raoultella* (ASV#4) | L-lysine | ath-miR158b | Exponential (0-32)  Stationary (33-52) | 0.001736  0.04073 | Negative |
|  | 17 L-AA mix | ath-miR158b | Exponential (0-10) | 0.03509 | Negative |
| *Acinetobacter* (ASV#6) | 17 L-AA mix | ath-miR5642b | Exponential (0-14) | 0.02326 | Positive |
|  | L-Phe | ath-miR158b | 24-45  46-52 | 0.04355  0.03668 | Positive |
|  |  | ath-miR5642b | 24-45 | 0.04480 | Positive |
|  |  | miRNA mix | 0-2  3-23 | 0.003491  0.00743 | Positive |
| *Chryseobacterium* (ASV#172) | 17 L-AA mix | ath-miR158b | Exponential (4-28) | 0.00128 | Negative |

The positive or negative impacts of plant miRNAs (*p* <0.05) on the main growth phases of cultures treated with the tetrazolium dye. Paired T-tests of the area under the curve of each growth phase were performed (n=5).

Table S9: Genes associated to the nitrogen cycle for each isolate.

|  | ***Acinetobacter*** | ***Chryseobacterium*** | ***Raoultella*** |
| --- | --- | --- | --- |
| Nitrogen Fixation | NA | NifU | NifS |
| Nitrification | NA | NA | hydroxylamine reductase |
| Denitrification | NirB  NirD  nitrous oxide reductase family maturation protein NosD | NA | -nitrate reductase subunit alpha, beta and gamma  -nitrate reductase molybdenum cofactor assembly chaperone --NirB  -NirD  - anaerobic nitric oxide reductase flavorubredoxin  -NorR |
| Anaerobic Ammonium Oxidation (Anammox) | NA | NA | NA |
| Dissimilatory Nitrate Reduction to Ammonium (DNRA) | NA | NA | NA |
| Ammonium Assimilation | -glutamine synthetase  -FMN-binding glutamate synthase  -glutamate synthase large subunit  -NADP-specific glutamate dehydrogenase | -glutamine synthetase III  -FMN-binding -glutamate synthase  folylpolyglutamate synthase/dihydrofolate synthase  -NADP-specific glutamate dehydrogenase | -glutamine synthetase  -glutamate synthase large & small subunits  -NADP-specific glutamate dehydrogenase |
| Ammonium transport | -ammonium transporter (3x) | -ammonium transporter | -AmtB ammonium transporter |
| Nitrogen Regulation | -nitrogen regulation protein NR(I)  -nitrogen regulation protein NR(II)  -P-II family nitrogen regulator | NA | -PTS IIA-like nitrogen regulatory protein PtsN  -nitrogen regulatory protein P-II |
| Nitrate/Nitrite Transport | formate/nitrite transporter | NA | -NarK nitrate/nitrite MFS transporter (2x)  -formate/nitrite transporter  -nitrite transporter  -nitrate ABC transporter permease |
| Amino Acid Transport | -branched-chain amino acid transport system II carrier protein  -aspartate-alanine antiporter  -GltP glutamate/aspartate:proton symporter  -ProP glycine betaine/L-proline transporter  -PutP sodium/proline symporter  -YddG aromatic amino acid DMT transporter  - SstT serine/threonine transporter  -YgaH L-valine transporter subunit | -branched-chain amino acid ABC transporter substrate-binding protein  -glycine betaine/L-proline ABC transporter ATP-binding protein  -MetIQ methionine ABC transporter | -LivKHMGF high-affinity branched-chain amino acid ABC transporter  -branched-chain amino acid ABC transporter permease (2x)  -L-methionine/branched-chain amino acid transporter  -branched-chain amino acid ABC transporter substrate-binding protein (2x)  -BrnQ branched-chain amino acid transporter carrier protein  -HisJPQ histidine ABC transporter permease  -p-aminobenzoyl-glutamate transporter -GltKP glutamate/aspartate:proton symporter  -ProY proline-specific permease  -ProPVWX glycine betaine/L-proline transporter  -PutP sodium/proline symporter  -AroP aromatic amino acid transporter  -aromatic amino acid transport family protein  -YddG aromatic amino acid DMT transporter (2x)  - cadaverine/lysine antiporter  -ArgT lysine/arginine/ornithine ABC transporter substrate-binding protein  -lysine-specific permease  -phenylalanine transporter  - DsdX D-serine transporter  - SstT serine/threonine transporter  -HAAAP family serine/threonine permease  -TdcC threonine/serine transporter  -tryptophan permease  -TyrP tyrosine transporter  -YgaH L-valine transporter subunit  -MetINQ methionine ABC transporter |
| General Amino Acid Transport | -amino acid ABC transporter permease (4x)  -amino acid ABC transporter substrate-binding protein (1x)  -amino acid ABC transporter ATP-binding protein (2x) | NA | -YbbAP putative ABC transporter permease subunit  -amino acid ABC transporter ATP-binding protein (7x)  -amino acid ABC transporter permease (12x)  -amino acid ABC transporter substrate-binding protein (2x)  -amino acid ABC transporter permease/ATP-binding protein (2x) |
| Oligopeptide Transport | -peptide MFS transporter  -SbmA peptide antibiotic transporter | -peptide MFS transporter (3x) | -OppABCF oligopeptide ABC transporter  -DppABCF  dipeptide ABC transporter  --peptide MFS transporter  -SbmA peptide antibiotic transporter |
| Molecules that increase plant AA efflux | - PhzF family phenazine biosynthesis protein (2x) | - PhzF family phenazine biosynthesis protein | - PhzF family phenazine biosynthesis (2x) |

Table S10: Predicted miRNA targets linked to amino acid transportation or nitrogen regulation.

| **Isolate** | **miRNA** | **Target** | **Blastn** | **miRanda** | **psRNATarget** | **IntaRNA** |
| --- | --- | --- | --- | --- | --- | --- |
| *Acinetobacter* | ath-miR159a | **contig_3_cds_pgaptmp_002450_2369** putP sodium/proline symporter | Position: 964-972 evalue: 0.67 | Position: 954-974 Energy: -14.64 kcal/mol | Position: 954-974 Expectation: 4.5 | Position: 964-972 Energy:-8.96 kcal/mol |
|  |  | **contig_3_cds_pgaptmp_002458_2377** sstT serine/threonine transporter | Position: 677-685 evalue: 0.67 | Position: 663-686 Energy: -19.33 kcal/mol | Position: 170-192 Expectation: 4.5 | Position: 664-685 Energy:-11.25 kcal/mol |
|  |  | **contig_3_cds_pgaptmp_003018_2936** glnG nitrogen regulation protein NR(I) | Position: 409-416 evalue: 2.6 | Position: 908-1019 Energy: -14.95 kcal/mol | Position: 396-420 Expectation:5 | Position: 399-422 Energy: -13.56 kcal/mol |
|  | ath-miR827 | **contig_3_cds_pgaptmp_002388_2308** AzlC family ABC transporter permease | Position 199-205 evalue: 10 | Position: 185-206 Energy: -6.30 kcal/mol | Position 185-206 Expectation: 3.5 | Position: 195-205 Energy: -7.75 kcal/mol |
|  | scramble ath-miR158a-3p | **contig_3_cds_pgaptmp_002304_2224** amino acid ABC transporter permease | Position: 140-147 evalue: 2.4 | Position: 276-295 Energy:-6.00 kcal/mol | Position 276-295 Expectation: 3.5 | Position: 270-279 Energy: -9.24 kcal/mol |
|  | scramble ath-miR827 | **contig_3_cds_pgaptmp_002975_2894** brnQ branched-chain amino acid transport system II carrier protein | Position: 422-428 evalue: 10 | Position: 414-433 Energy: -20.94 kcal/mol | Position: 414-433 Expectation: 4 | Position: 422-432 Energy:-9.43 kcal/mol |
|  |  | **contig_3_cds_pgaptmp_003288_3206** proP glycine betaine/L-proline transporter | Position: 510-518 evalue: 0.67 | Position: 495-518 Energy: -10.90 kcal/mol | Position: 495-518 Expectation: 2 | Position: 502-518 Energy: -10.29 kcal/mol |
| *Chryseobacterium* | scramble ath-miR158b | **contig_1_cds_pgaptmp_001027_1008** metQ methionine ABC transporter substrate-binding lipoprotein | Position: 165-176 evalue:0.49 | NA | Position:164-183 Expectation: 1.5 | Position: 165-179 Energy: -11.79 kcal/mol |
|  |  |  |  |  |  |  |
| *Raoultella* | ath-miR158b | **contig_1_cds_pgaptmp_004439_4327** proY proline-specific permease | Position: 373-379 evalue: 34 | Position: 361-380 Energy: -12.30 kcal/mol | Position: 82-101 Expectation: 4.5 | Position: 373-379 Energy: -6.87 kcal/mol |
|  | ath-miR827 | **contig_1_cds_pgaptmp_002844_2750** abgT p-aminobenzoyl-glutamate transporter | Position: 336-346 evalue: 0.14 | Position: 85-105 Energy: -11.86 kcal/mol | Position 85-105 Expectation: 2.5 | Position: 91-104 Energy: --6.06 kcal/mol |
|  |  | **contig_1_cds_pgaptmp_002984_2890** amino acid ABC transporter/ATP-binding protein | Position: 214-222 evalue: 8.7 | Position: 209-232 Energy: -15.20 kcal/mol | Position 206-232 Expectation: 2 | Position: 214-230 Energy: -8.66 kcal/mol |
|  |  | **contig_1_cds_pgaptmp_001739_1676** amino acid ABC transporter permease/ATP-binding protein | Position: 300-306 evalue: 34 | Position: 211-231 Energy: -11.14 kcal/mol | Position: 211-231 Expectation: 5 | Position: 224-230 Energy: -6.96 kcal/mol |
|  |  | **contig_1_cds_pgaptmp_002732_2641** amino acid ABC transporter permease/ATP-binding protein | Position: 214-222 evalue: 2.2 | Position: 211-231 Energy: -21.13 kcal/mol | Position: 211-231 Expectation: 4.5 | Position:215-230 Energy: -8.06 kcal/mol |
|  | ath-miR5642b | **contig_1_cds_pgaptmp_000221_211** proP glycine betaine/L-proline transporter | Position: 1389-1395 evalue: 34 | Position: 1377-1396 Energy: -17.97 kcal/mol | Position: 390-413 Expectation: 4.5 | Position: 1389-1395 Energy: -6.66 kcal/mol |
|  |  | **contig_1_cds_pgaptmp_003523_3429** yddG aromatic amino acid DMT transporter | Position: 58-52 evalue: 34 | Position: 63-84 Energy: -22.12 kcal/mol | Position: 63-84 Expectation: 3 | Position: 66:83 Energy: -11.24 kcal/mol |
|  |  | **contig_1_cds_pgaptmp_001168_1118** mtr tryptophan permease | Position: 573-579 evalue: 34 | Position: 367-386 Energy: -24.99 kcal/mol | Position: 367-386 Expectation: 4.5 | Position: 372-385 Energy: -5.06 kcal/mol |
|  | scramble ath-miR158a-3p | **contig_1_cds_pgaptmp_001168_1118** mtr tryptophan permease | Position: 863-870 evalue: 8.7 | Position: 74-93 Energy: -12.88 kcal/mol | Position: 852-870 Expectation: 4 | Position: 856-870 Energy: -8.41 kcal/mol |
|  |  | **contig_1_cds_pgaptmp_004488_4376** amino acid ABC transporter ATP-binding protein | Position: 14-21 evalue: 8.7 | Position: 2-21 Energy: -12.59 kcal/mol | Position: 2-21 Expectation: 3.5 | Position: 49-57 Energy: -5.87 kcal/mol |

The light grey highlights binding sites at a different position of the CDS compared to the other tools. Position: position along the contig where a significant match was found; evalue: BLAST e-value, evaluating the chance of alignments with the same score or better; energy: sum of the energy of the hybridization and the energy required to unfold the interaction sites in both RNA molecules, se refs. 75-77; expectation: psRNATarget score, as described in ref. 74.

Supplementary figures


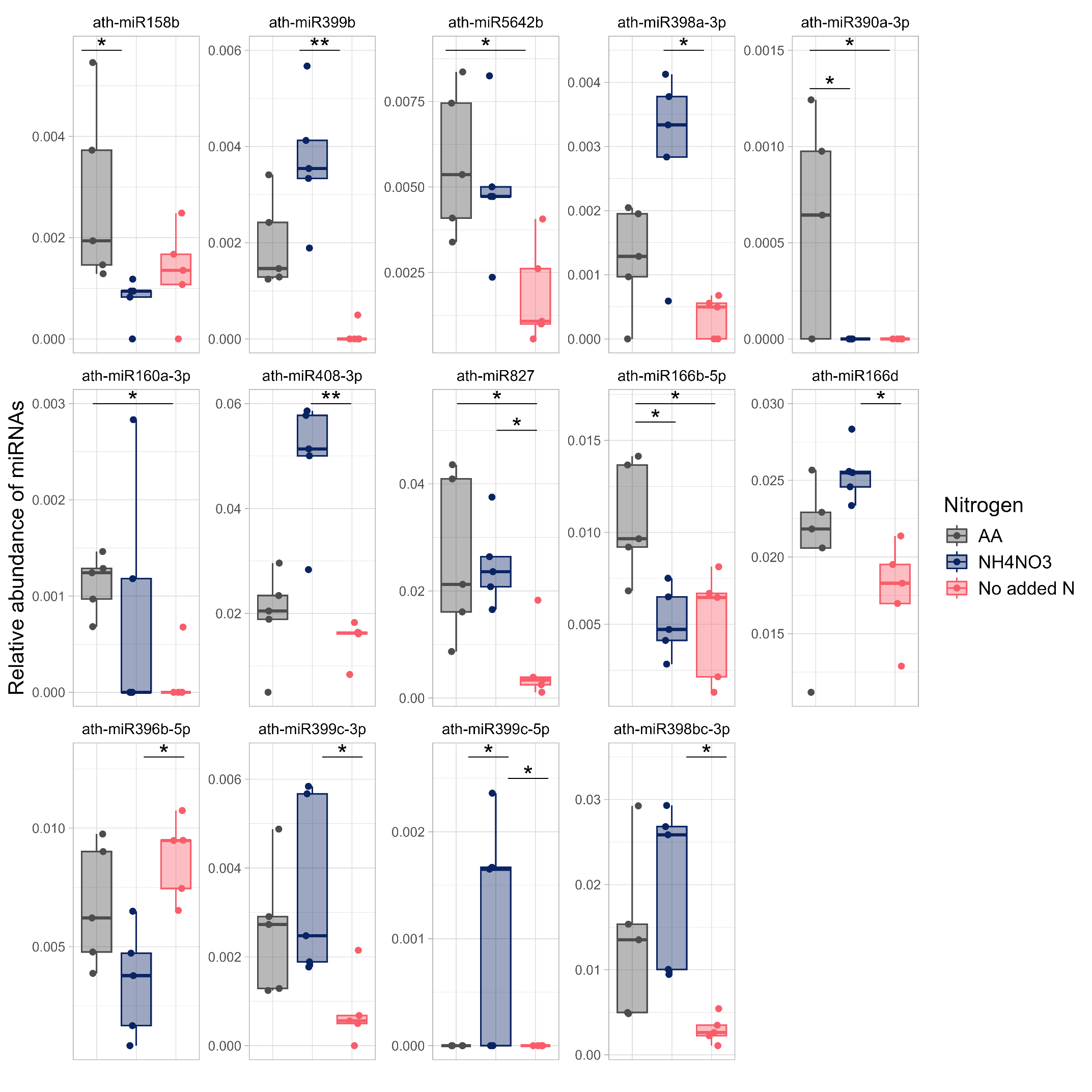


Figure S1: The relative abundance of N-responding root miRNAs. Differences in relative abundance of miRNAs between N treatments are indicated with brackets (*p*- adjusted with Holm correction <0.05, n=5).


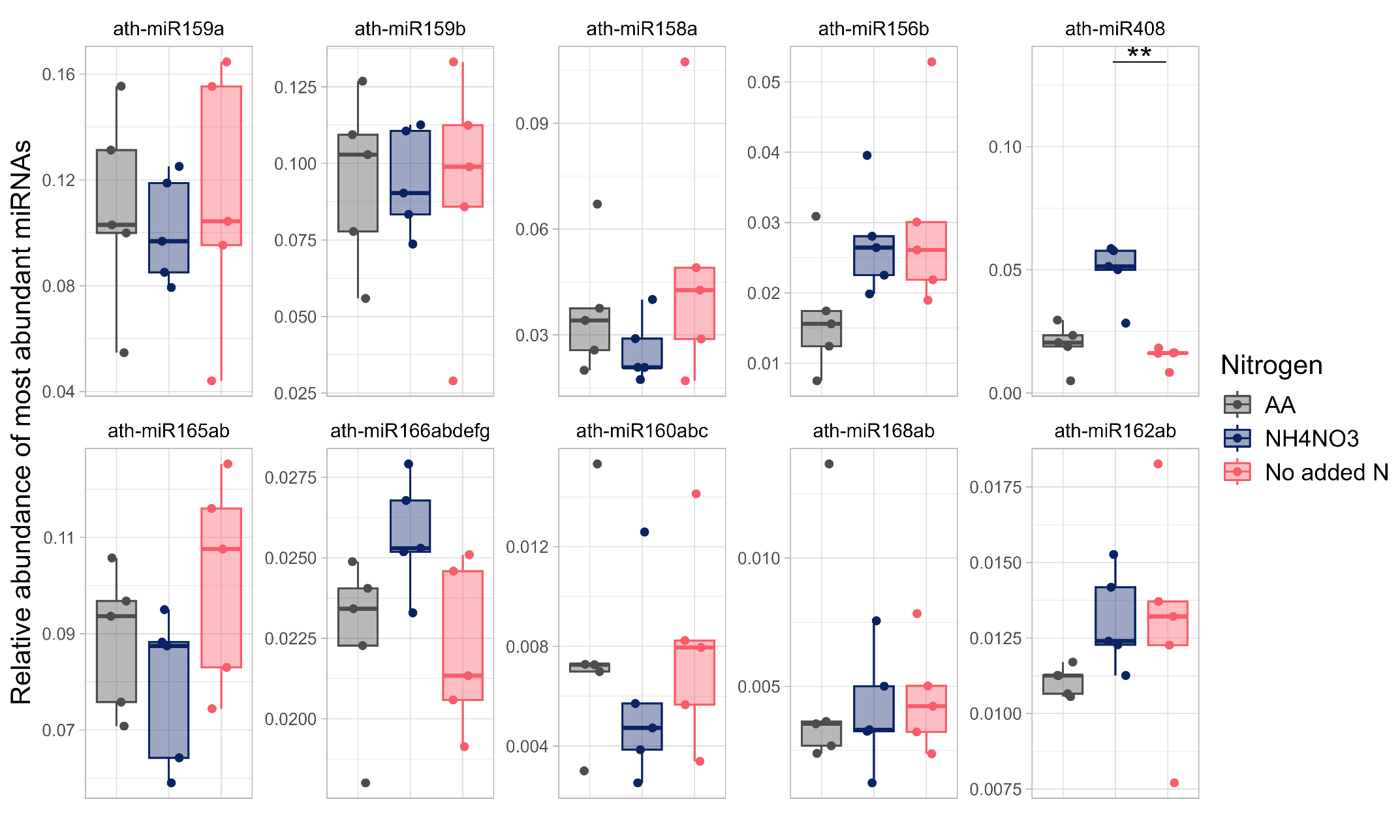


Figure S2: The relative abundance of the most abundant root miRNAs. Differences in relative abundance of miRNAs between N treatments are indicated with brackets (*p*- adjusted with Holm correction <0.05, n=5).


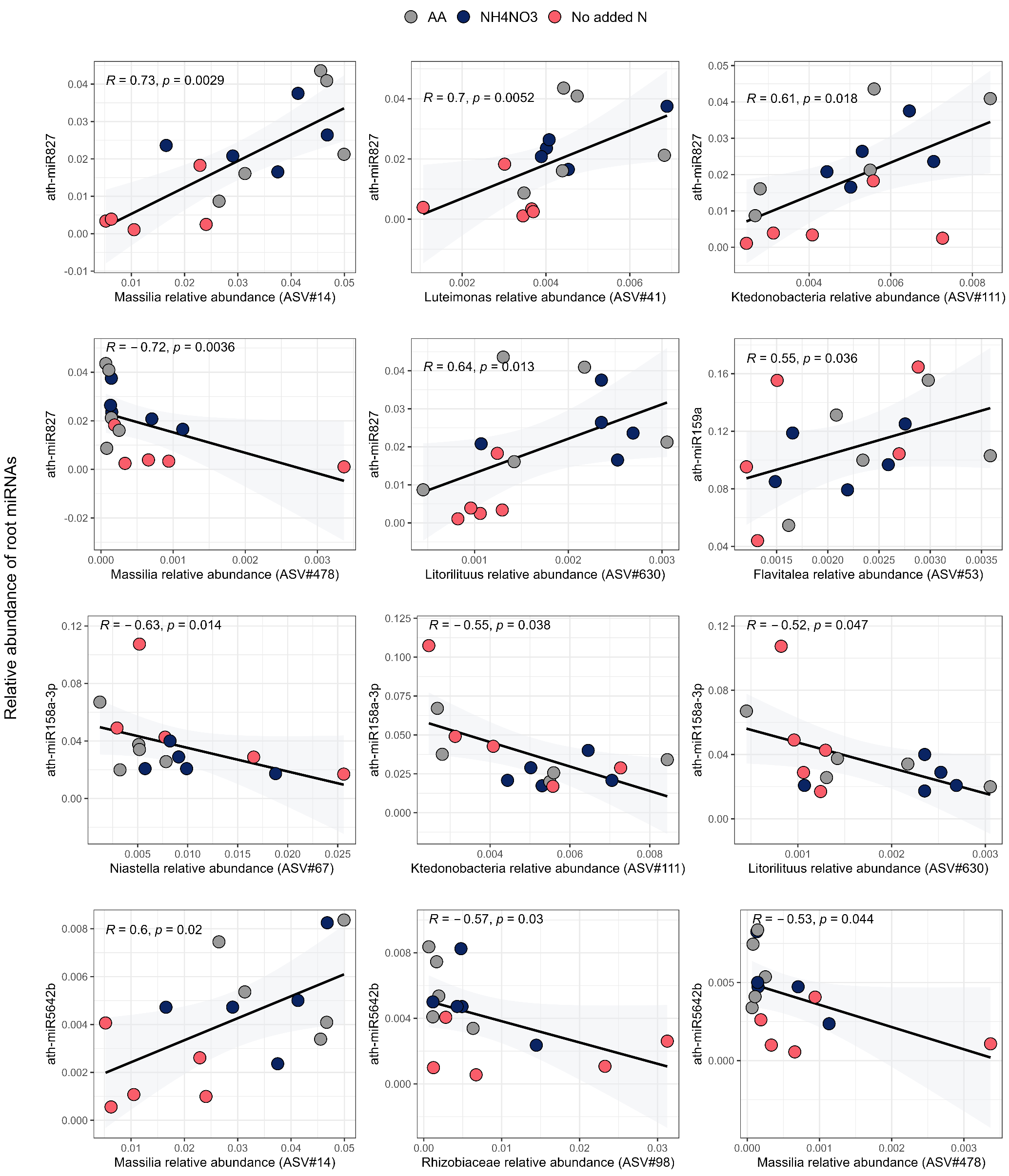


Figure S3: Spearman correlations between the relative abundance of our miRNAs of interest and bacterial ASVs (16S rRNA gene). No significant correlations were identified with ath-miR158b (*p*<0.05, n=5).


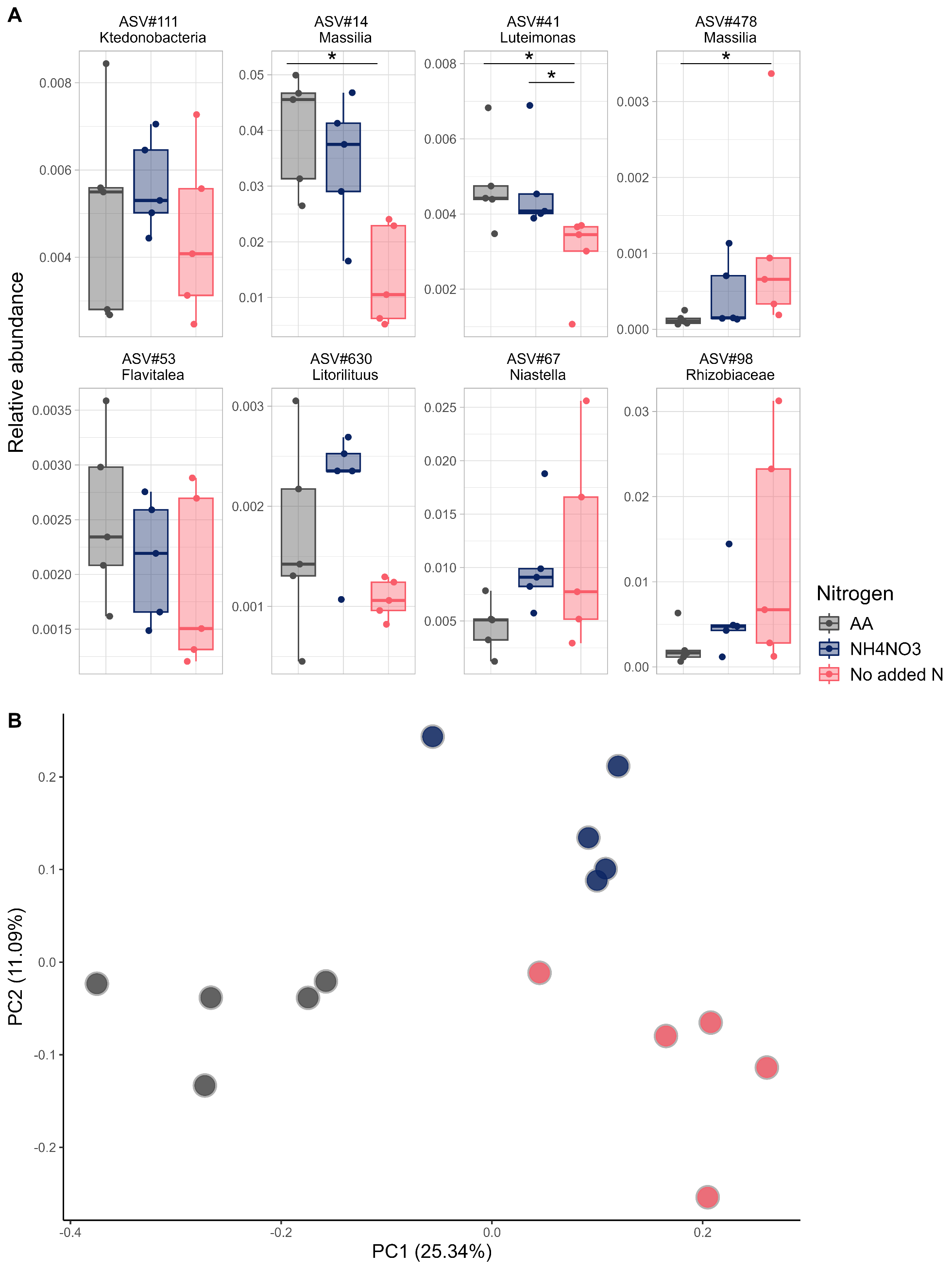


Figure S4: Bacterial taxa (16S rRNA gene) in the roots of *A. thaliana*. A. relative abundance of ASVs depending on the nitrogen treatment. Differences in relative abundance of taxa between N treatments are indicated with brackets (Dunn’s test with a Benjamini-Hochberg correction *p*<0.05, n=5). B. Principle coordinate analysis that must be interpreted with the first principle component only (n=5).


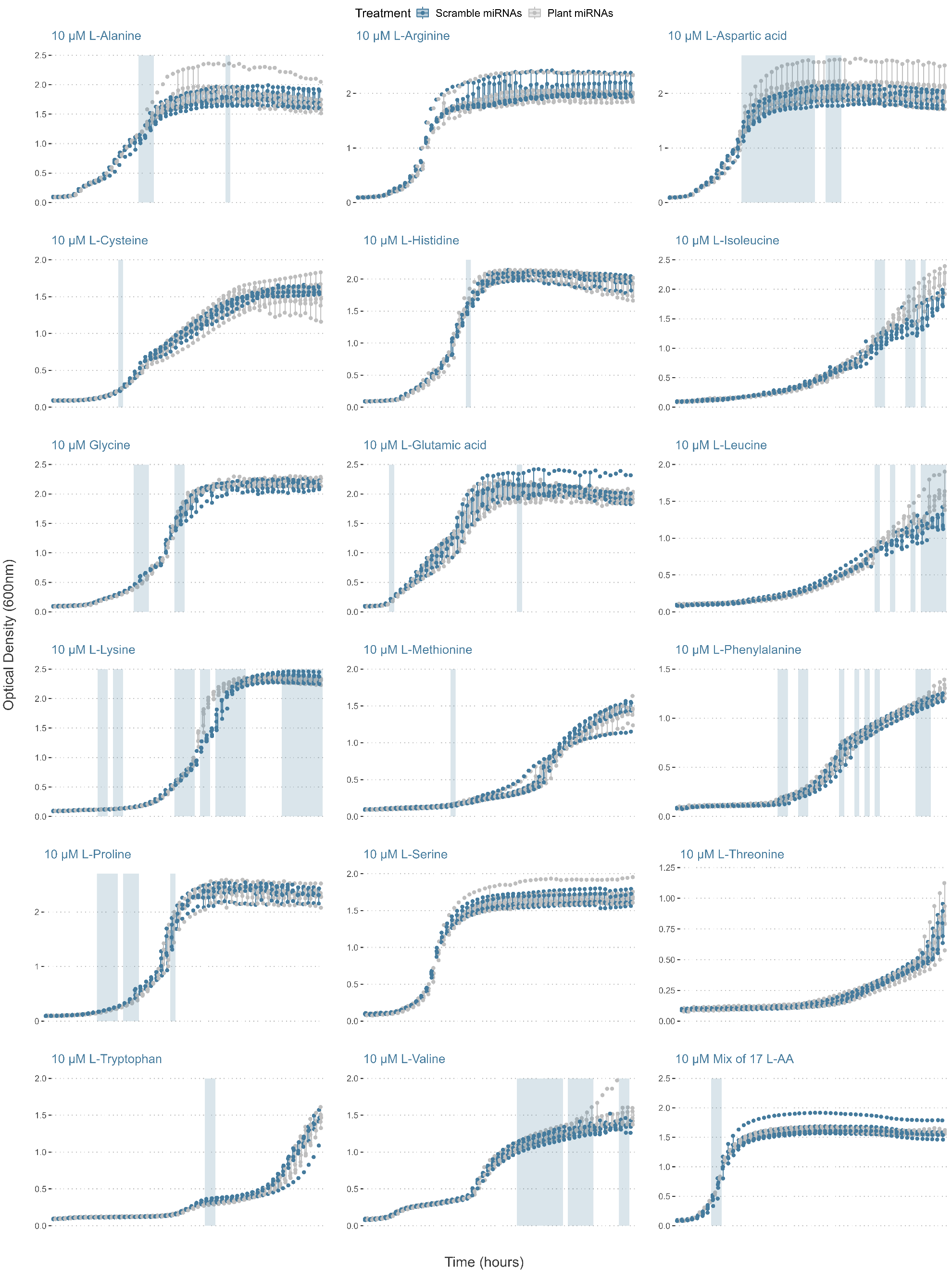


Figure S5: Growth curves, enhanced by the addition of a tetrazolium dye, of soil microbes (n=5) grown with different amino acids as a nitrogen source. The highlighted parts of the growth curve indicate differences *p*<0.05 in optical density (600 nm) of microbes treated with the mix (10 µM) plant miRNAs compared to their respective scrambled miRNAs (paired T-test, n=5).


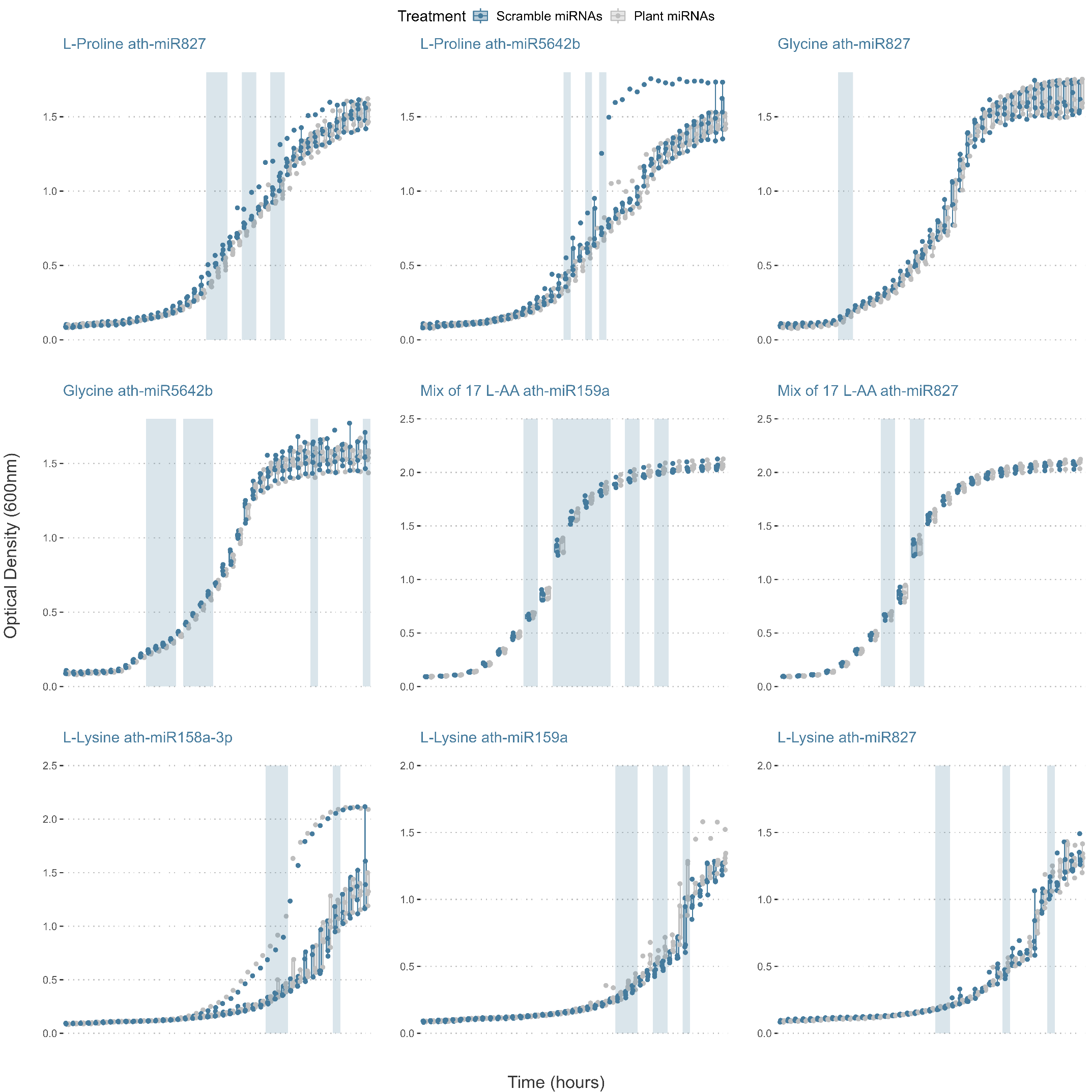


Figure S6: Growth curves, enhanced by the addition of a tetrazolium dye, of soil microbes (n=5) grown with different amino acids as a nitrogen source. The highlighted parts of the growth curve indicate differences *p*<0.05 in optical density (600 nm) of microbes treated with a single (2 µM) plant miRNA compared to its respective scrambled miRNA (paired T-test, n=5).


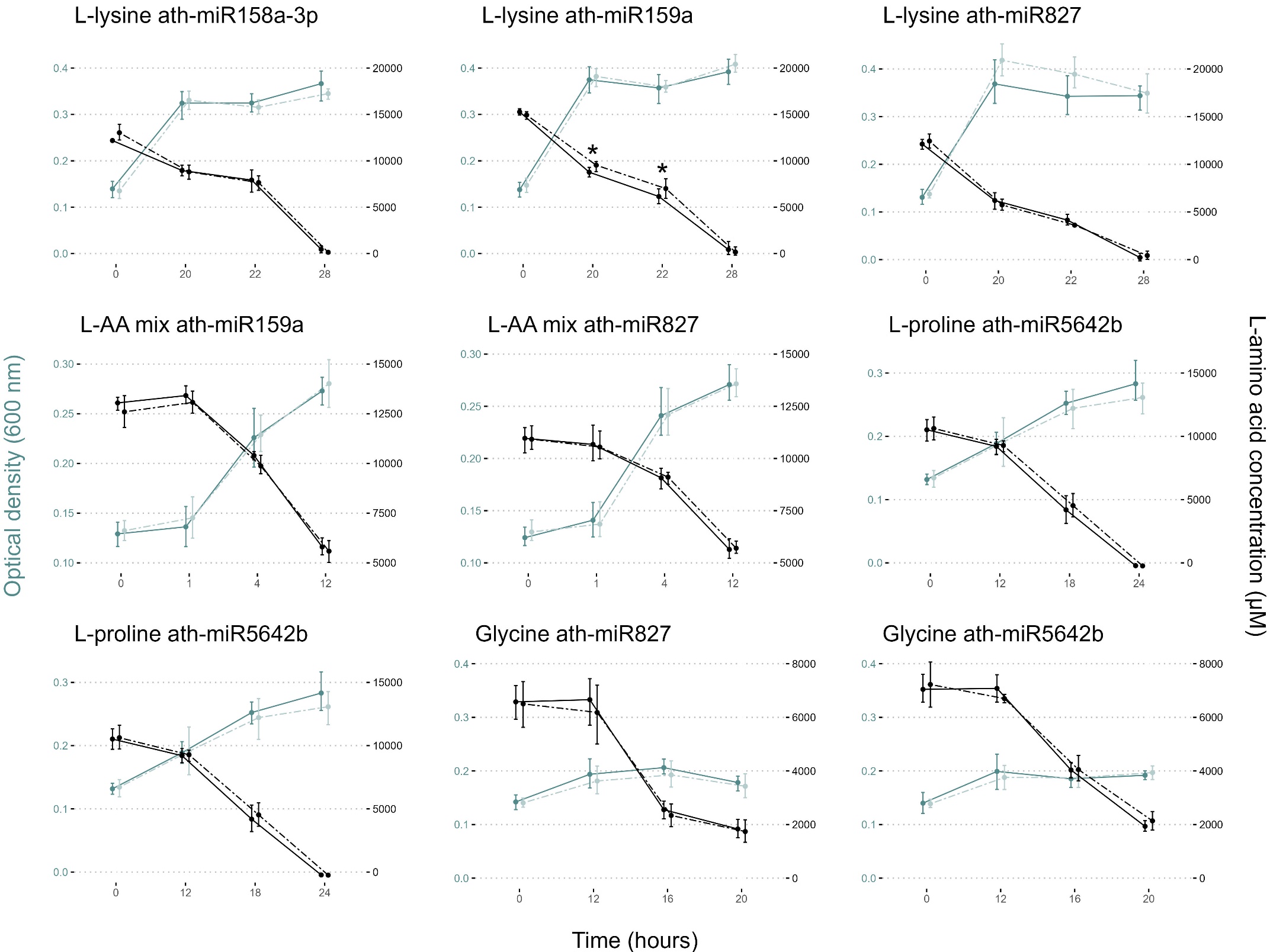


Figure S7: Microbial growth (OD600, blue lines) and amino acid consumption (black lines) over time (hours). Dashed lines indicate that the microbes were exposed to 2 µM of plant miRNAs whereas full lines indicate that the microbes were exposed to 2 µM of corresponding scrambled miRNA. Significant results are identified with a * (*p*<0.05 paired T-test) (n=5).


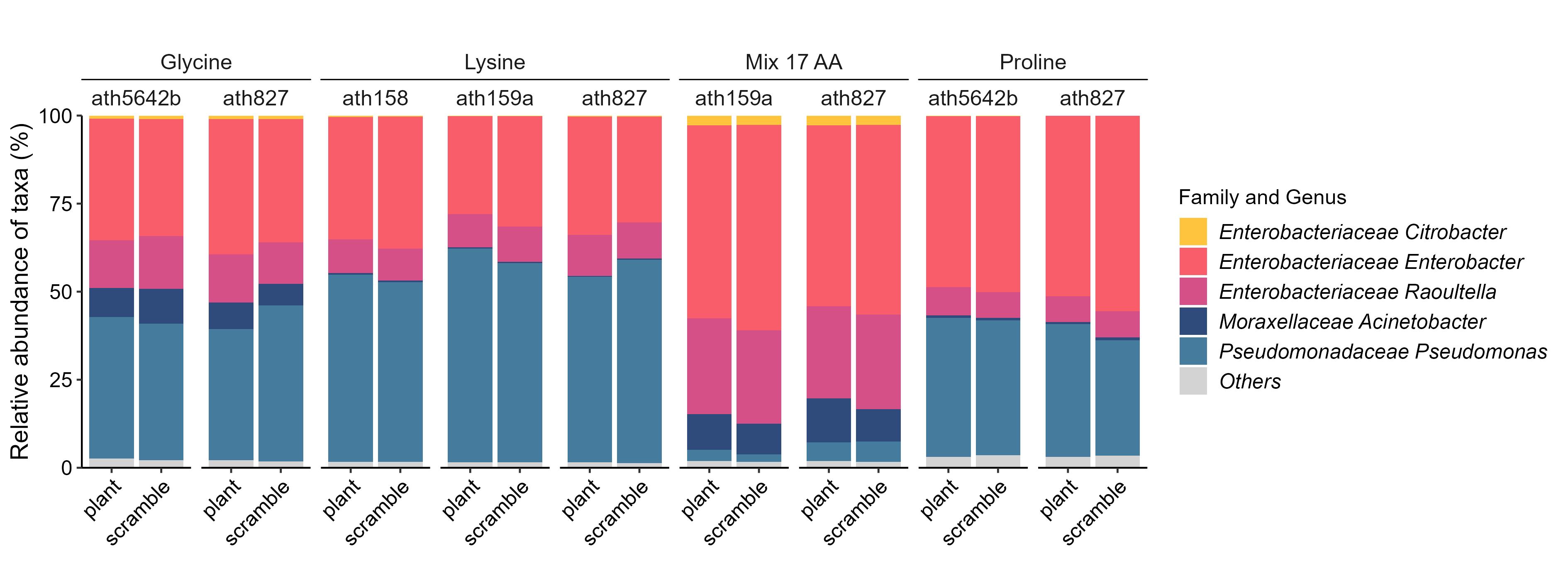


Figure S8: Mean relative abundance of bacterial taxa (16S rRNA gene) exposed to 2 µM of miRNAs (plant or scrambled) in four different amino acid sources (n=5).


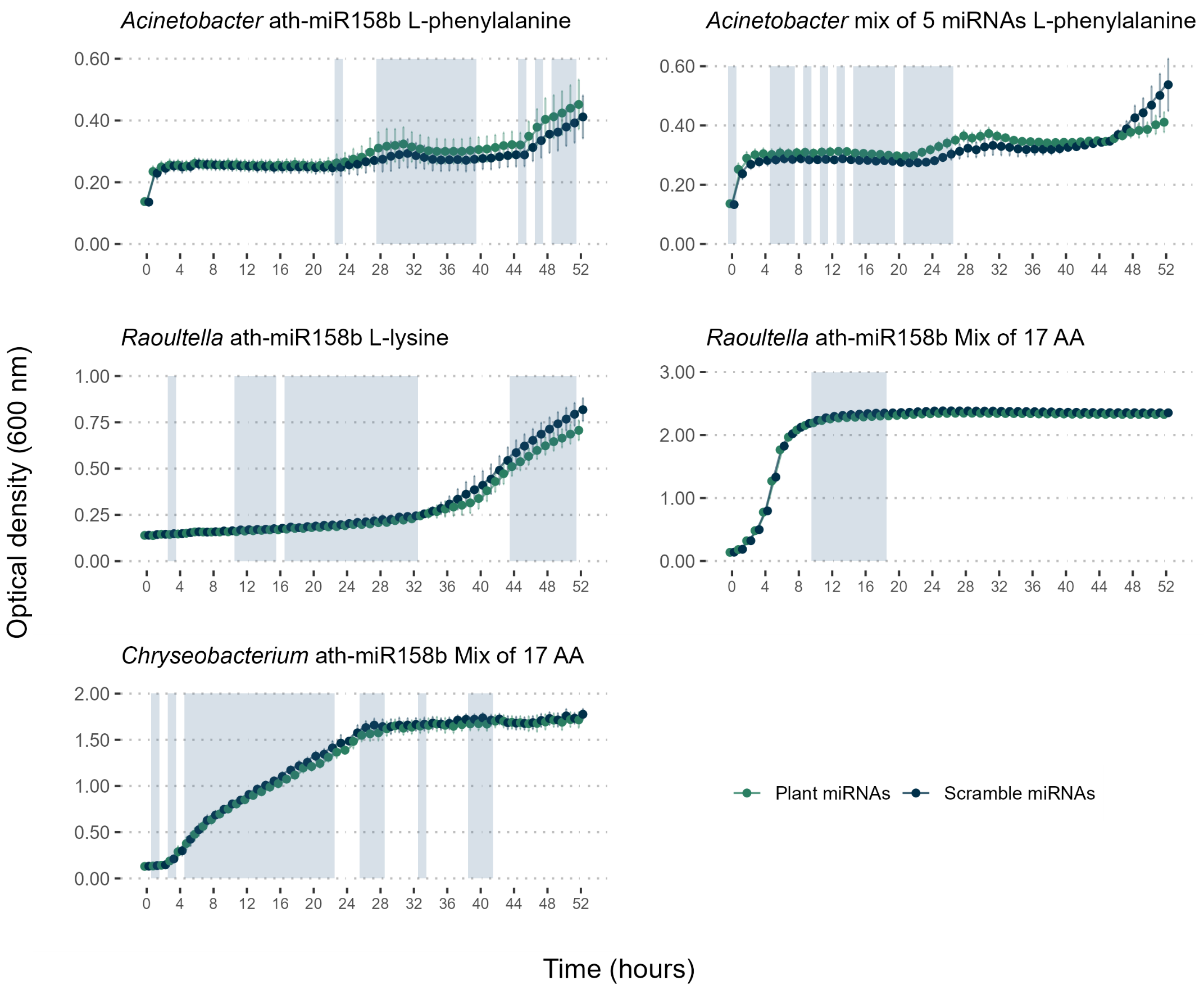


Figure S9: Growth curves of the isolates exposed to miRNAs. The highlighted parts of the growth curve indicate differences *p*<0.05 in optical density (600 nm) of isolates treated with plant miRNAs compared to the scrambled miRNA (paired T-test or paired Wilcox test) (n=5).
